# Sequential Effect of Dual‐Layered Hybrid Graphite Anodes on Electrode Utilization During Fast‐Charging Li‐Ion Batteries

**DOI:** 10.1002/advs.202403071

**Published:** 2024-06-13

**Authors:** Jiwoong Kang, Jaejin Lim, Hyuntae Lee, Seongsu Park, Cheol Bak, Yewon Shin, Hyeongguk An, Mingyu Lee, Minju Lee, Soyeon Lee, Byungjun Choi, Dongyoon Kang, Sujong Chae, Yong Min Lee, Hongkyung Lee

**Affiliations:** ^1^ Department of Energy Science and Engineering Daegu Gyeongbuk Institute of Science and Technology (DGIST) 333 Techno Jungang‐daero, Hyeonpung‐eup, Dalseong‐gun Daegu 42988 Republic of Korea; ^2^ Department of Industrial Chemistry Pukyong National University 45 Yongsoro Busan 48513 Republic of Korea; ^3^ Department of Chemical and Biomolecular Engineering Yonsei University 50 Yonsei‐ro, Seodaemun‐gu Seoul 03722 Republic of Korea; ^4^ Energy Science and Engineering Center DGIST 333 Technojungang‐daero Daegu 42988 Republic of Korea

**Keywords:** dual‐layered electrodes, fast‐charging batteries, hybrid graphite anodes, resistance distribution, temporal SOC gradient

## Abstract

To recharge lithium‐ion batteries quickly and safely while avoiding capacity loss and safety risks, a novel electrode design that minimizes cell polarization at a higher current is highly desired. This work presents a dual‐layer electrode (DLE) technology via sequential coating of two different anode materials to minimize the overall electrode resistance upon fast charging. Electrochemical impedance spectroscopy and distribution of relaxation times analysis revealed the dynamic evolution of electrode impedances in synthetic graphite (SG) upon a change in the state of charge (SOC), whereas the natural graphite (NG) maintains its original impedance regardless of SOC variation. This disparity dictates the sequence of the NG and SG coating layers within the DLE, considering the temporal SOC gradient developed upon fast charging. Simulation and experimental results suggest that DLE positioning NG and SG on the top (second‐layer) and bottom (first‐layer), respectively, can effectively reduce the overall resistance at a 4 C‐rate (15‐min charging), demonstrating two times higher capacity retention (61.0%) over 200 cycles than its counterpart with reversal sequential coating, and is higher than single‐layer electrodes using NG or NG/SG binary mixtures. Hence, this study can guide the combinatorial sequence for multi‐layer coating of various active materials for a lower‐resistivity, thick‐electrode design.

## Graphical abstract

A dual‐layered, hybrid anode design is proposed to maximize electrode utilization during fast‐charging lithium‐ion cells by leveraging the distinct impedance trends of natural and synthetic graphite anodes as the state of charge (SOC) changes. Combining experiments and simulation studies unlocks the sequential role of the active materials in the dual‐layer coating, considering the temporal SOC distribution that develops during fast‐charging.

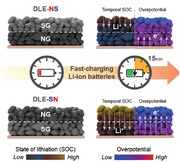

## 1 Introduction

As the widespread adoption of electric vehicles (EVs) hinges on charging time, current lithium (Li)‐ion batteries (LIBs) encounter new challenging goals—high energy density and fast‐charging capability.^[^ ^1^, ^2^, ^3^, ^4^, ^5^ ^]^ Besides exploring new materials, cutting‐edge cell engineering, including cell geometry, assembly, and electrode design, has made remarkable progress in enhancing energy density. Notably, electrode thickening the electrodes has become mandatory for EV‐targeted cell design.^[^ ^6^, ^7^, ^8^ ^]^ Instead of laborious implantation of high‐energy active materials, the fabrication of thick electrodes with conventional active materials can be a straightforward approach for enhancing the energy density by reducing weight and volume fractions of inactive components in the cell, including separators, current collectors, and casing/packaging. However, thicker electrodes can adversely increase cell polarization owing to the long and highly tortuous Li^+^ travel distance, as well as the extended electrical pathway, compromising high‐power capability,^[^ ^9^, ^10^ ^]^ thus highlighting the need to develop an innovative electrode platform to overcome the trade‐off relationship between energy density and fast‐charging capability.

Graphite anodes have long been employed in LIBs because of their low potential (<0.2 V vs Li/Li^+^), excellent Li^+^ reversibility, and low material price.^[^ ^11^ ^]^ Nonetheless, their thickening is considered the major origin of significant cell polarization during fast charging.^[^ ^12^, ^13^ ^]^ As thicker coating from the conventional slurry coating process induces considerable migration of polymeric binder in the electrode during evaporation of the solvent in the slurry, thicker electrodes are vulnerable to uneven distribution of carbon‐binder domains and hinder Li^+^ diffusion across the electrode.^[^ ^14^, ^15^ ^]^ Such a structural irregularity jointly enhances the overpotential, accidentally exceeding the Li plating potential (≈0 V vs Li/Li^+^).^[^ ^16^, ^17^ ^]^ Moreover, the thicker electrode can trigger an inconsistent degree of lithiation in a depth direction, i.e., inhomogeneous state‐of‐charge (SOC) in‐between graphite particles,^[^ ^18^, ^19^ ^]^ resulting in a local imbalance of the N/P ratio, which further triggers unintended Li plating and fatal loss of Li inventory, thereby resulting in substantial cell failure and safety concerns.^[^ ^20^, ^21^ ^]^

To overcome these drawbacks, novel electrode architectures have been proposed by creating Li^+^ transport channels and/or blending the different active materials. For instance, the laser‐guided micropatterning of as‐manufactured electrodes can provide well‐aligned Li^+^ bypass channels with near‐zero tortuosity.^[^ ^22^, ^23^ ^]^ However, cell capacity is inevitably sacrificed by removing pre‐existing active materials. As a solution, building a hybrid anode by mixing graphite and hard carbon can compensate for this capacity loss.^[^ ^24^ ^]^ Contrary to highly crystalized graphite, percolation of hard carbon particles can act as faster Li^+^ diffusion channels benefiting from its disordered graphene sheets, efficiently enhancing the cycling performance at high charging rates (≈6 C, ≈15.5 mA cm^−2^). When incorporating plenty of hard carbon, however, achieving a high energy density is still challenging owing to its poor initial CE (<80%) from Li^+^ trapped in the microporous structure and surficial side reaction with electrolyte from its functional group. Furthermore, given the relatively low true density (1.6 g cc^−1^; 2.2 g cc^−1^ for graphite) and the gradual voltage profile within the 0–1.2 V range, there is a disadvantage in achieving higher energy density, making a purposeful electrode design essential.

Apart from these efforts, a slurry coating using a two‐layer slot die enables a dual‐layered electrode (DLE) structure^[^ ^25^, ^26^ ^]^ involving different binder contents, yielding a better binder distribution in the electrode after the drying process. With this merit in the structural stability of the electrode, DLE can substantially enhance cycling performance under high‐rate conditions. As an early phase of the multilayer electrode coating technology, various parameters for DLE, such as the optimal composition of each layer, viscosity of slurries, etc., are actively under development. It is noteworthy that the multilayer coating enables the implementation of advanced electrode architecture tailored to specific applications (high‐power, high‐energy, and long‐cycle life), ensuring the advantageous distribution of electrode components. Nonetheless, selection criteria for the active materials have not yet been established when combining multiple materials, especially for attaining the fast‐charging capability.

This work presents a rational selection of active materials and their sequential combinations for DLE fabrication. We compared two different graphites—synthetic (SG) and natural graphite (NG)—using electrochemical impedance spectroscopy (EIS) and distribution of relaxation times (DRT) analysis. By leveraging distinct differences in their SOC‐deterministic impedance evolutions, overall electrode impedances in the DLE can be intelligently reduced upon the temporal SOC gradient by repositioning the NG and SG on the top (near‐separator) and bottom (near‐current collector), respectively. Notably, the SG‐NG sequentially coated DLE (DLE‐SN) outperforms its counterpart (DLE‐NS) and is even superior to single‐layer electrodes (SLEs) using only NG and NG/SG mixtures. The sequential effect in the DLE framework was cross‐validated by a postmortem analysis and a simulation study via pseudo‐3D (P3D) modeling.

## 2 Results and Discussion

**Figure 1:**
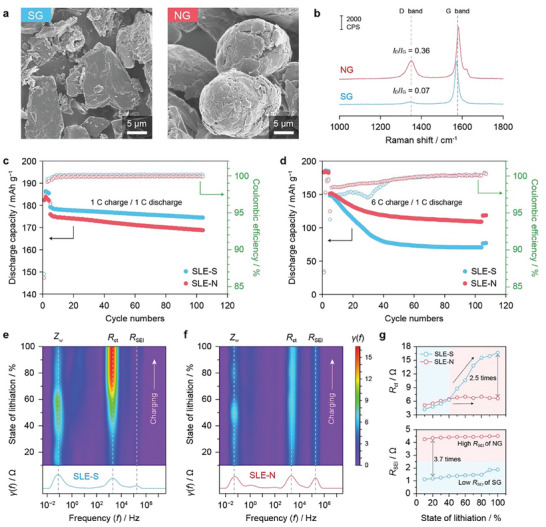
Preliminary comparison of NG and SG. a) SEM images and b) Raman spectra for NG and SG particles. c) Capacity retentions of graphite||NMC622 full cells using NG and SG‐based single‐layer electrodes (SLE‐N and SLE‐S, respectively) at 1 C charge/discharge (=1.9 mAh cm^−2^). d) Cycling test under fast‐charging conditions (at 6 C charge and 1 C discharge). In‐situ DRT analysis for e) SLE‐S and f) SLE‐N during charging. g) Charge transfer resistance (*R*_ct_) and solid‐electrolyte interphase (SEI) resistance (*R*_SEI_) variation with respect to the state‐of‐lithiation (i.e., SOC).

**Figure** **1**a shows the morphological contrast between SG and NG materials; SG is flake‐like, while NG is spherical, and SG features a pore‐less structure, while NG has many pores inside, allowing more electrolyte penetration (Figure S1, Supporting Information). Through the particle size analyzer (PSA), the average particle sizes (*D*_50_) of SG and NG are confirmed as 14 and 17 µm, respectively (Figure S2, Supporting Information). In the Raman analysis (Figure 1b), the G band at ≈1580 cm^−1^ represents the in‐plane vibrations of the carbon atoms in graphene layers, while the D band at ≈1350 cm^−1^ reveals disordered arrangements in the graphite surface.^[^ ^27^, ^28^ ^]^ The intensity ratio (*I*_D_/*I*_G_) of the two peaks exhibit 0.36 and 0.07 for NG and SG, respectively, indicating that NG possesses a more disordered structure and can provide more diffusion channels of Li^+^ than SG. Additionally, the adsorptive potential distributions from nitrogen adsorption allow the quantitative analysis of the surface heterogeneity,^[^ ^29^, ^30^ ^]^ dividing into basal and nonbasal plane surfaces, which accumulate edge and defective surfaces (Figure S3, Supporting Information). The surface area of each plane and its fraction can be calculated using a modified nonlocal density functional theory (MNLDFT) (Table S1, Supporting Information). Considering that the intercalation of Li^+^ ion occurs predominantly through the nonbasal plane, NG can be advantageous for fast‐charging over SG because of its higher fraction of the nonbasal plane (16%) than SG (9%).

X‐ray photoelectron spectroscopy (XPS) spectra obtained after the formation cycle reveal the differences in solid‐electrolyte interphase (SEI) components on the surfaces of NG and SG (Figure S4, Supporting Information). Notably, NG exhibits a reduced intensity of C 1s peaks and Li_x_PO_y_F_z_ peak while LiF peak is pronounced, suggesting that an intensive SEI‐forming reaction occurs via lithium hexafluorophosphate (LiPF_6_) hydrolysis,^[^ ^31^, ^32^, ^33^ ^]^ likely due to the abundance of highly reactive defects on the edge‐plane‐rich NG surfaces (Figure S3, Supporting Information). In the electrochemical aspect, the specific capacity of NG (360 mAh g^−1^) is higher than that of SG (340 mAh g^−1^), as confirmed in a Li||graphite half‐cell testing (Figure S5, Supporting Information), while the graphite||LiNi_0.6_Mn_0.2_Co_0.2_O_2_ (NMC622) full cell with SG‐containing SLE (SLE‐S) exhibited slightly better capacity retention (98.7%) than the cell with NG‐based SLE (SLE‐N) over 100 cycles (97.2%) at a modest C‐rate (1 C) (Figure 1c). Besides its larger surface area (Figure S6, Supporting Information), the substantial volume change in NG can trigger continuous SEI formation at the SLE‐N anode during repeated cycling,^[^ ^34^, ^35^, ^36^ ^]^ resulting in a loss of Li inventory and, consequently, lower capacity retention compared to the SLE‐S anode. When applying a higher current (6 C, ≈11.4 mA cm^−2^), the trend reversed (Figure 1d): while the initial capacities were similar (145 and 141 mAh g^−1^ for SLE‐S and SLE‐N), the SLE‐S cell suffered from rapid capacity decay by 72 mAh g^−1^ (49% retention). In contrast, the SLE‐N cell retained a relatively higher capacity 100 mAh g^−1,^ and 71% retention even after 100 cycles. Therefore, compared to SG, the sphere‐shaped, largely disordered NG is beneficial to fast‐charging LIBs despite the less stability upon long cycling at a moderate current.

To further investigate the electrochemical features, we conducted an EIS analysis by varying the SOC (Figure S7, Supporting Information). Interestingly, the EIS spectra of the NG did not change regardless of the SOC, while the overall impedance of the SG significantly increased as the SOC increased. The DRT method offers a precise frequency‐domain resolution, allowing for the clear identification and characterization of individual resistance components. Each polarization process can be associated with various time constants by applying the DRT technique.^[^ ^37^, ^38^ ^]^ The DRT study revealed that SG (SLE‐S) features lower SEI resistance (*R*_SEI_) than NG (SLE‐N), and its charge transfer resistance (*R*_ct_) increases as the SOC increases (Figure 1e). In contrast, NG exhibited a relatively higher *R*_SEI_ (4.50 Ω) than the counterpart (1.89 Ω), but both *R*_SEI_ and *R*_ct_ remained constant regardless of SOC (Figure 1f). The higher *R*_SEI_ of SLE‐N is likely attributed to extensive side reactions owing to the abundance of defects on the NG surfaces.^[^ ^39^, ^40^ ^]^ After SOC 40%, the *R*_ct_ of SG rapidly increased from 4.25 Ω (SOC 0%) to 8.79 (SOC 50%) and 16.17 Ω (SOC 100%) (Figure 1g). Given that the SOC gradient can be developed across the anodes at a higher charging current, the electrode resistance of the SLE‐S will be heterogenous owing to the temporal SOC gradient: top surface (near the separator), where the SOC reaches 100% earlier, is more resistive than the bottom (near the current collector). In contrast, SLE‐N can maintain relatively lower overpotentials, benefiting from SOC‐independent *R*_SEI_ and *R*_ct_, alleviating Li plating at the top region. In other words, while SG could be beneficial at a lower SOC (<30%) owing to its lower *R*_SEI_ than NG, the NG is more advantageous in reducing the electrode resistance at a higher SOC (>40%). A comparison of the Li^+^ diffusion coefficient (*D*_Li+_) using the galvanostatic intermittent titration technique (GITT) showed that NG has a higher diffusion coefficient than SG (Figures S8 and S9, Supporting Information), suggesting that NG can facilitate the Li^+^ intercalation kinetics. In this regard, the positioning of the SG and NG within the DLEs, considering the difference, could influence their performance during fast‐charging cycles.

**Figure 2:**
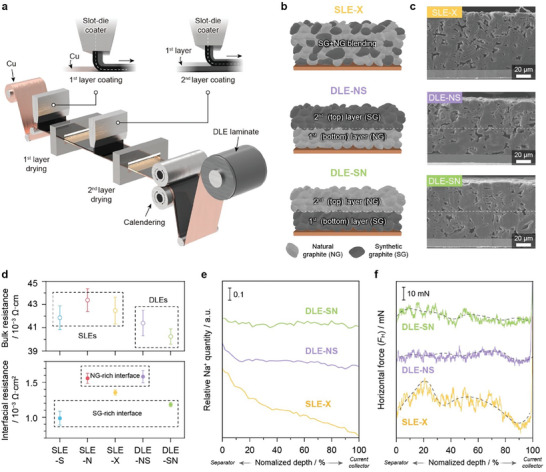
Electrode structures and binder distributions at SLEs and DLEs. Schemes of a) DLE fabrication process and b) as‐proposed SLE‐X, DLE‐NS, and DLE‐SN hybrid anodes. c) FIB‐SEM images of as‐prepared SLE‐X and DLEs. d) Selective measurement of interface and bulk resistances of SLEs and DLEs using the electrode resistance measurement system. e) Depth profiles of relative sodium (Na^+^) counts during Na‐LIBS. f) Depth profiling of horizontal forces (*F*_H_) measured at the SAICAS micro‐blade for SLE‐X and DLEs.

In addition to SLE‐N and SLE‐S, SLE‐X was fabricated by blending NG and SG (50:50 by weight) for comparison. To manufacture the DLEs (**Figure** **2**a), we repeated the electrode‐slurry coating/drying processes twice to prevent mixing each slurry layer with identical viscosities and solid contents at the bottom and top layers. To reveal the sequential effects of the active materials on the DLE structures, we fabricated two types of DLEs (Figure 2b): NG coated on the bottom layer (near‐current collector) and SG coated on the top layer (near‐separator) (DLE‐NS) and SG coated on the bottom layer and NG coated on the top layer (DLE‐SN). By investigating the cross‐sectional images of the electrode through FIB‐SEM (Figure 2c), the distribution of NG and SG can be observed based on the porosity of graphite particles and their structural features (Figure S1, Supporting Information). For a clear distinction between NG and SG, the image segmentation is presented in Figure S10 (Supporting Information). From the image segmentation, the borderline of DLE‐NS and DLE‐SN can be clearly observed, which is in the middle of the electrode. It is noted that this image analysis corresponds well to the composition of DLE‐NS and DLE‐SN electrodes, where the DLE‐NS electrode is composed of 48% NG and 52% SG, while the DLE‐SN electrode is comprised of 49% SG and 51% NG (Figure S11, Supporting Information). Then, we selectively measured the bulk and interface (electrode‐current collector) resistances of the as‐prepared electrodes using an electrode resistance measurement system (Figure 2d). As both SLE‐N and DLE‐NS share the NG‐rich interfaces, they exhibited comparable interface resistances (1.5–1.6 mΩ cm^2^). Likewise, the DLE‐SN with SG‐rich interface showed a similar value with the SLE‐S (1.0–1.1 mΩ cm^2^). These results demonstrate that the two DLEs were well‐fabricated in different orders. Accordingly, SLE‐X represents the intermediate value (≈1.3 mΩ cm^2^), suggesting that the SG and NG were well‐mixed. Unlike the interface resistance, the electrode‐bulk resistance suggests that the SLE exhibited a comparatively higher than the DLEs, possibly attributed to the uniformity of the binder distribution.

When manufacturing thick electrodes, a challenge arises as the binder tends to self‐migrate toward the solvent vaporizing direction during the drying process. This migration deteriorates the uniformity of the electrode and leads to increased resistance.^[^ ^41^, ^42^ ^]^ The binder distribution was further evaluated by Na^+^‐laser‐induced breakdown spectroscopy (LIBS) since Na^+^ is an indicator of the CMC binder location. While the SLE‐S and SLE‐N consistently showed a decrease in the relative quantity of Na^+^ in the depth direction (Figure 2e; Figure S12, Supporting Information), the DLEs showed an overall even distribution. Furthermore, through surface and interfacial cutting analysis system (SAICAS) analysis (Figure 2f),^[^ ^43^, ^44^ ^]^ we confirmed the variation in the binder‐governed cohesion by gradually moving the blade in the electrode depth direction and selectively measuring the horizontal forces (*F*_H_). SLE‐X showed a significant *F*_H_ in the top region but continuously decreased as it descended toward the Cu current collector. Similar fluctuations were observed in both SLE‐S and SLE‐N (Figure S13, Supporting Information). In contrast, both DLEs consistently exhibited negligible changes in *F*_H_ across the electrodes. This examination of the binder distribution and its local binding characteristic suggests that stepwise coating of each layer upon DLE fabrication can improve the binder distribution, even for thick electrodes. Therefore, DLE can address the uneven binder distribution for thick‐electrode manufacturing, and a comparison of SG and NG coating sequences within DLEs can be possible in the material base by ensuring binder uniformity.

**Figure 3:**
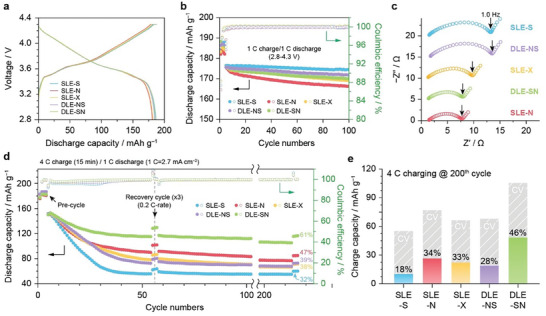
a) Voltage profiles of graphite||NMC622 full cells containing SLEs and DLEs at stabilization cycle. b) Cycle performances of graphite‖NMC622 full cells (2.7 mAh cm^−2^, N/P = 1.1) at a 1 C‐rate (=2.7 mA cm^−2^). c) Nyquist plots of graphite||graphite symmetrical cells with harvested anodes after charging up to SOC 50% at a 4 C‐rate. d) Cycle performances of graphite||NMC622 full cells (2.7 mAh cm^−2^, N/P = 1.1) at 4 C in the constant‐charging‐constant voltage (CC‐CV) charging mode (15‐min time cut‐off). e) Comparison of CC and CV charge capacity at 200^th^ cycles during 15‐min at 4 C‐rate.

In an electrochemical aspect, we performed the graphite||NCM622 full‐cell tests with the SLEs and DLEs. Although the voltage profiles during pre‐cycling were almost consistent (**Figure** **3**a), there was a slight loss of the initial CEs at the NG‐containing anodes owing to the large surface area and inherent higher reactivity at the edge plane. Indeed, the SLE‐S cell still exhibits the highest capacity retention (98.9%) after 100 cycles at 1 C charge/discharge (Figure 3b). Nonetheless, SLE‐X (96.8%) and DLEs (98.4 and 97.1% for DLE‐NS and DLE‐SN, respectively) are better than solely use of NG (SLE‐N, 95.4% retention) since NG‐triggered side reactions can be partially alleviated. Therefore, benefiting from even binder distributions, DLE can less compromise the performance standard at modest C‐rates than that of SLE‐X. Deviating from the modest C‐rate, we compared the electrode impedances in the graphite||graphite symmetric cell configuration after precycle (Figure 3c). Notably, each anode was harvested from the full cells, which were precharged to SOC 50% at a 4 C‐rate. Among the SLEs, the interfacial resistances declined in the order SLE‐S > SLE‐X > SLE‐N, suggesting that increasing the NG content helps reduce electrode polarization despite the slight capacity loss. Although both DLEs have the same NG content, DLE‐SN exhibited a much lower interfacial resistance than DLE‐NS and even outperformed SLE‐X, indicating that the preferential location of NG within the hybrid anodes is the top surface, which contributes to a reduction in electrode polarization.

To evaluate the fast‐charge capability, a constant current (CC)‐constant voltage (CV) charging protocol with a charging time cut‐off was used (15‐min for 4 C, ≈11 mA cm^−2^), following the DOE battery‐testing guidelines, the retentions of total cell capacity during 4 C cycling were compared for SLEs and DLEs (Figure 3c). As confirmed previously, a steep capacity drop was observed in the SLE‐S cell, and the available capacity did not recover, showing poor retention (32.1%) at the 200^th^ cycle, attributed to significant Li plating during fast charging. On the contrary, the capacity retention of the SLE‐N cell (46.8%) still outperformed that of the SLE‐X and DLE‐NS cells (38.0 and 39.0% retention, respectively), demonstrating that the introduction of SG particles is not very beneficial in enhancing fast‐charging cycling. Nevertheless, the DLE‐SN cell exhibited diminished capacity degradation over the initial 50 cycles at 4 C, indicative of a significant reduction in Li plating. This reduction is evidenced by the notably enhanced capacity retention (61.0%) at the same 200 cycles, surpassing that of the SLE‐N cell, despite the constrained charging duration. In the selected voltage profiles at the 25^th^, 100^th^, and 200^th^ cycles, DLE‐SN exhibited a longer CC charging period than the other anodes (Figure S14, Supporting Information), indicating that cell polarization was further suppressed. It is worth comparing the capacity contribution of the CC charging mode (Figure 3d) to confirm lower cell polarization and achieve higher capacity retention.^[^ ^22^, ^45^, ^46^ ^]^ Indeed, the SLE‐S cell showed the lowest CC‐charging capacity (18%) and immediately turned into the CV‐charging mode, which is inefficient for attaining the capacity within the limited 15‐min charging time (Figure 3e). The CC‐charging capacity was substantially improved in the early stage for NG‐containing cells but rapidly dropped after 200 cycles, where the portions of CC‐charging capacity are 34, 33, and 28% for SLE‐N, SLE‐X, and DLE‐NS, respectively. However, DLE‐SN exclusively well‐retained a higher portion of the CC‐charging capacity (46% at the 200^th^ cycle), suggesting that the position of the NG and SG particles must be carefully determined for successful NG‐SG hybridization through the DLE framework beyond SLE‐N and SLE‐X. Therefore, DLE‐SN can effectively reduce cell polarization, maintaining a lower impedance upon fast‐charging cycling, thereby achieving a higher capacity through the greater contribution of CC charging.

**Figure 4:**
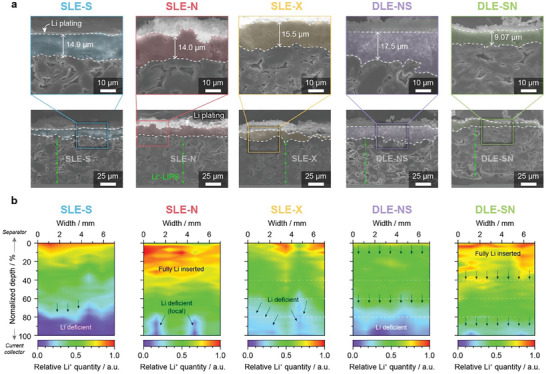
Postmortem analysis. a) FIB‐assisted cross‐sectional SEM images harvested after 50 cycles at 4 C charging rate. b) Li^+^‐LIBS‐guided Li^+^ distribution maps for SLEs and DLEs harvested after 1^st^ 4 C charging.

After 50 cycles under fast charging conditions, the Li plating thicknesses were compared by cross‐sectional SEM images of the five different anodes (**Figure** **4**a). The DLE‐SN, which showed the best capacity retention, exhibited the minimum Li plating (9.1 µm), while the DLE‐NS suffered from extensive Li plating (17.5 µm). Similarly, other SLEs showed a thicker Li plating layer (≈15 µm). It is noteworthy that the DLE‐SN can effectively suppress unwanted Li plating even better than solely using NG (SLE‐N). Interestingly, we confirmed that Li plating is possibly preferred on the NG surface owing to the abundance of surface defects. The spatial distribution of the Li deposits was determined from the composition of NG and SG at the top surface of the SLEs and DLEs obtained after 10 cycles (Figure S15, Supporting Information). Interestingly, while the SLE‐N and DLE‐SN allowed Li plating evenly, the SLE‐S and DLE‐NS exhibited sporadic Li deposition. Thus, positioning NG in the top region further helps mitigate local Li dendrite growth by spreading out the Li deposits, even when Li plating inevitably occurs.

To evaluate electrode utilization, Li^+^‐LIBS was performed to reveal the local Li^+^ concentration map across after lithiation at 4 C‐rate (Figure 4b). Given that Li plating occurred in all the cases, the distribution maps were normalized by the maximum Li^+^ quantity of plated Li at the topmost surface. For SLE‐S, which showed the worst performance, most of the Li inserted was concentrated in the top region; however, lithiation hardly occurred at the bottom part, indicating poor electrode utilization. In contrast, Li^+^ insertion in SLE‐N and SLE‐X was substantially facilitated but mostly occurred in the top region, and local Li^+^ deficiency at the bottom was still confirmed. Notably, DLE‐NS showed the worst electrode utilization, showing no 100% SOC region, explaining the occurrence of heavy Li plating. However, DLE‐SN efficiently utilizes the entire electrode, exhibiting a higher Li concentration without a Li^+^‐deficient region. Given the electrode mass loading and testing protocol, Li plating is unavoidable with the conventional electrolyte (1.15 m LiPF_6_ in ethylene carbonate/ethyl methyl carbonate (EC/EMC), 3:7 v/v with 2 wt.% vinylene carbonate (VC)), which is outdated for fast‐charging applications. Nonetheless, SEM and LIBS depth profiling revealed that DLE‐SN can effectively alleviate Li plating by maximizing electrode utilization upon fast charging, leading to improved capacity retention over prolonged cycling. Furthermore, Li plating can be eliminated by successfully integrating DLE‐SN with advanced electrolytes featuring high ionic conductivity,^[^ ^47^, ^48^ ^]^ a higher Li^+^ transference number,^[^ ^49^, ^50^ ^]^ and a low‐resistance SEI.^[^ ^51^, ^52^ ^]^ To further validate the benefit of DLE‐SN, an advanced electrolyte (3 m LiPF_6_ in DMC +5 wt.% FEC) was employed for XFC cycling (Figure S16, Supporting information), which can facilitate Li^+^ desolvation and promote the formation of thinner, anion/additive‐derived SEI.^[^ ^53^ ^]^ DLE‐SN consistently showed higher capacity retention (96.4%) after 50 cycles than that of DLE‐NS (94.8%), which demonstrates further reduction of cell polarization through efficient electrode utilization in DLE‐SN.

**Figure 5:**
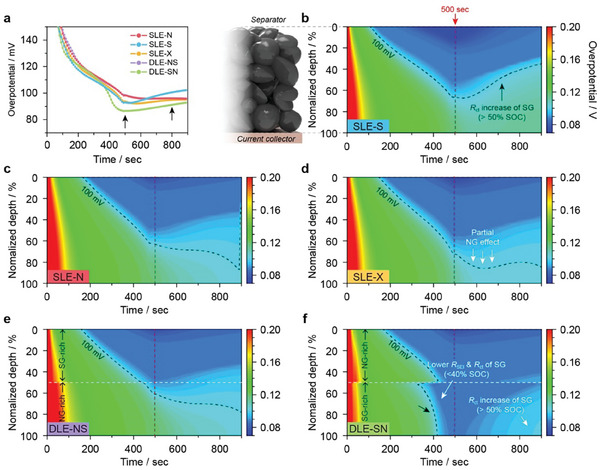
a) Transient overpotential responses of SLEs and DLEs within 15‐min charging at 4 C‐rate. Black arrows indicate remarkable differences in temporal overpotential between each anode. Time‐sequential overpotential (TSO) distribution maps across the anode depth (normalized by total thickness, ≈72 µm) for b) SLE‐S, c) SLE‐N, d) SLE‐X, e) DLE‐NS, and f) DLE‐SN anodes. All data were extracted from the COMSOL simulation results.

To provide mechanistic insights into the exceptional improvement in the fast‐charging capability of the DLE‐SN anode, P3D models for a graphite||NMC622 cell were constructed, with COMSOL simulations performed to reflect the CC‐CV charging protocol. Additional details regarding the models (Figure S17, Supporting Information) and equations (Table S2, Supporting Information), parameters (Tables S3–S5, Supporting Information), and validation (Figure S18, Supporting Information) are described in the Supporting Information. Notably, we focused on the temporal and spatial variations in the SOC throughout the anode thickness and the consequences of this heterogeneity on electrode polarization. Considering the interplay between the electrode impedances and SOC, we first derived time‐resolved SOC distribution maps (Figure S19, Supporting Information), verifying that positioning the NG particles in the top region facilitates lithiation and increases the SOC during the fast‐charging process. Nonetheless, a severe SOC gradient was initiated after 150 s in all the cases. **Figure** **5**a presents the time‐resolved overpotential changes across the SLEs and DLEs during 4 C‐rate charging. Upon initiating charging, all the cells exhibited a high overpotential (>200 mV) owing to the ohmic resistance, which rapidly decreased within 300 s. However, after 500 s, the overpotential of SLE‐S ramped up exceeding 120 mV during prolonged charging, likely due to a rapid *R*_ct_ increase. SLE‐N and DLE‐NS exhibit similar overpotential evolutions, with a modest increase in the overpotential during the later stages of charging. Similarly, incorporating NG into SLE‐X effectively mediated the overpotential increase during the subsequent charging process. In contrast, the overpotential of DLE‐SN further decreased to 88 mV after 500 s, maintaining a lower overpotential throughout the entire charging process.

Figures 5b–f provide the time‐sequence overpotential (TSO) maps for each anode to examine the spatial and temporal variations in the overpotentials throughout the anode thickness. Although the overpotentials at the top region consistently lowered in all cases, a SOC‐driven disparity in the overpotential was observed after 500 s. SLE‐S retains a high local overpotential (>100 mV) in the bottom‐half region (Figure 5b; Figure S20a, Supporting Information), SLE‐N can effectively alleviate the overpotential during prolonged charging (Figure 5c). Compared with SLE‐S, the TSO map of SLE‐X exhibited prompt SOC increases in the deeper region—responsible for the earlier reduction in the overpotential after 500 s (Figure 5d). Despite the prompt lithiation in the bottom region of the DLE‐NS, the reduction in overpotential was less and delayed up to 700 s (Figure 5e). In contrast, the TSO map for DLE‐SN demonstrated a reduced overpotential in the lower half of the region (Figure 5f). Even as a temporal SOC gradient developed during a later stage of charging, the SG positioned where subjected to lower SOC levels region outmatched the NG in maintaining lower resistance. Even toward later charging (≈800 s), the SG at the bottom layer of the DLE‐SN retained a 12.5% lower overpotential than that of the SLE‐N (Figure S20b, Supporting Information), benefiting from the lower *R*_SEI_ at a lower SOC, leading to a further reduction in the electrode resistance. These findings confirm that location‐specific coating of the active materials within the DLEs can engineer the overall electrode polarization even under fast‐charging conditions. Therefore, DLE‐SN can contribute to redistributing the electrode resistances via SOC gradient‐guided mediation, improving the electrode utilization even at fast‐charging C‐rates, ultimately resulting in a higher capacity and reduced cell polarization.

**Figure 6:**
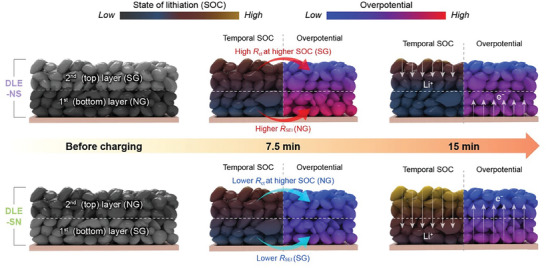
Schemes of sequential effect in DLE‐NS and DLE‐SN hybrid anodes on electrode utilization during the fast‐charging process. The SOC gradient represents an actual color change of graphite particles. The spatial and temporal overpotential distributions within each anode are illustrated according to the TOC map results.

While the precise determination of the SOC variation across the electrodes and by individual particles remains challenging,^[^ ^54^, ^55^ ^]^ the higher local current density at the graphite particles near the anode/separator interface can promote lithiation compared with the rest of the anode, resulting in a SOC gradient along the anode thickness, thereby leading to a highly inhomogeneous overpotential distribution (**Figure** **6**). When the surface of the SG particles becomes fully saturated with Li, they become more susceptible to Li plating as charging continues.^[^ ^56^, ^57^, ^58^ ^]^ Therefore, strategically placing NG particles in the topmost layer of the anode is beneficial owing to their prompt lithiation, even during high‐current charging. Besides, owing to the low resistance (*R*_SEI_) of SG in the low SOC, it is advantageous to the alleviation of the overpotential to locate SG at the bottom of the electrode. Despite electrolyte modification being more effective in enhancing XFC performance rather than electrode restructuring, the temporal SOC gradient effect could be more severe in thick electrodes. Therefore, the DLE design principle proposed in this study can help reinforce XFC capability for high‐energy cell design by incorporating advanced electrolytes. Consequently, it is imperative to delve deeper into the compositional effects of the NG/SG ratio within DLE‐SN to ensure the long‐term stability of Li‐ion battery cycling. Furthermore, additional validation is necessary with thicker electrodes and diverse materials such as silicon/graphite composites or nongraphitic materials (e.g., hard carbon and graphene derivatives). To address inherent limitations in Li^+^ diffusion, novel graphite structures, such as multi‐layered graphene,^[^ ^59^, ^60^ ^]^ expanded graphite,^[^ ^61^, ^62^ ^]^ graphene‐like graphite^[^ ^63^, ^64^ ^]^ could be a possible solution by offering larger interlayer spacing, facilitating enhanced Li^+^ diffusion. Moreover, advanced electrode additives, such as carbon nanotubes and graphene, could be selectively formulated in each layer to compensate for the disparity of locally different kinetics in the SOC gradient.^[^ ^65^, ^66^, ^67^ ^]^ As analogous phenomena are anticipated on the cathode side, the proposed principles for combinatorial active materials through sequential DLE coating can be readily extended to binary cathode systems that exhibit SOC‐dependent behavior. This extension holds promise for further mitigation of electrode polarization and minimization of cell polarization.

## 3 Conclusion

In summary, we achieved fast‐charging LIBs using DLE‐SN and revealed the effect of the coating sequence of the NG and SG layers on reducing electrode‐driven polarization. Apart from SLEs, DLEs exhibit unique merits in terms of uniform binder distribution upon thick electrode fabrication. While NG has been considered for enhancing the rate capability, SG is still preferred for securing long‐term cycling stability owing to its low reactivity toward the electrolyte. Although blending NG and SG is rational to exploit both advantages, the exposure of SG at the topmost surface, which features a drastic *R*_ct_ increase at a higher SOC, is not preferred. Therefore, SLE‐N and DLE‐SN can alleviate accidental Li plating and enhance electrode utilization, even when a severe SOC gradient in the electrode depth direction can temporarily develop at high current charging (>4 C). As confirmed in the simulation‐guided TSO map analysis, DLE‐SN positioned the SG at the bottom layer, which has a much lower *R*_SEI_ than NG, further reducing the electrode resistance compared to SLE‐N, eventually leading to improved capacity retention and less deterioration of the graphite surfaces upon fast‐charging cycling. Hence, this study establishes guidelines for DLE manufacturing to determine the locations of different active materials toward fast‐recharging, thick electrodes, overcoming the energy and power density tradeoffs in LIBs.

## 4 Experimental Section

### Electrode Fabrication

The SLEs and DLEs comprised active materials, conductive materials (Super P, Imerys), and a binder (50:50 wt.% sodium of carboxymethyl cellulose (Na‐CMC) and styrene‐butadiene rubber (SBR) mixture) in a weight ratio of 94:3:3 based on deionized water to prepare a well‐dispersed slurry. Active materials—SG (S360) and NG (SG17)—were purchased from BTR. To fabricate DLE‐NS and DLE‐SN, SG and NG slurries were prepared separately. The first‐layer slurry is cast using a doctor blade onto 10 µm copper (Cu) foils (Iljin, I2B). The cast slurries were dried at 60 °C for 2 h. The second‐layer slurry was then coated onto the pre‐existing first layer using the same process. The resulting hybrid anodes were pressed to the target electrode density using a roll press machine. For comparison, SLE‐S, SLE‐N, and SLE‐X were fabricated by conventional single‐layer coating using the same method. For all anodes, the mass loadings were controlled to attain the same areal capacity (≈3 mAh cm^−2^), with the electrode density fixed at 1.3 g cc^−1^.

### Cell Assembly

All the cathodes were prepared using NMC622, Super P, and a polyvinylidene fluoride (PVDF) binder in a weight ratio of 93:4:3. The slurry was cast onto aluminum (Al) foils (15 µm) and dried at 120 °C for 2 h. Then, the cathodes were calendered to attain the targeted electrode density (2.8 g cc^−1^). The final areal capacity is set to 2.7 mAh cm^−2^, and the N/P ratio corresponds to 1.1–1.15. Before the cell assembly, the as‐prepared cathode and anodes were cut into Ø14 and Ø16 mm disks, respectively, and vacuum‐dried at 60 °C for 12 h. Coin‐type (CR2032) graphite||NMC622 full cells were assembled in an Ar‐filled glove box by combining the cathodes and each anode using a polyethylene (PE) separator (Ø19 mm, Tonen). For the liquid electrolyte, 1.15 m LiPF_6_ was dissolved in a mixture of EC/EMC, 3:7 volume ratio with 2 wt.% VC additive. All chemicals were qualified as battery grade and purchased by Enchem (South Korea). The electrolyte (75 µL) was injected for all electrochemical testing.

### Electrochemical Measurements

Before the main cycling test, all cells were aged for 12 h. After the cell pre‐aging, the cells were preliminarily cycled for four cycles, including one formation cycle and three stabilizing cycles, in the voltage range of 2.8–4.3 V at 25 °C using a battery cycler (WBCS3000L, Wonatech). For the formation step, the cells were charged and discharged at 0.1 C in CC mode. During the stabilization step, the cells were charged at 0.2 C in the CC mode and continuously charged under CV mode after reaching 4.3 V. Then, the cells were subsequently discharged at 0.2 C via CC mode. This cycling protocol was repeated three times. For the fast‐charging cycling protocol, cells were evaluated at 25 °C, following the DOE battery‐testing guidelines.^[^ ^22^, ^24^ ^]^ For 4 C‐rate charging cycling, the CC‐CV charging period was fixed at 15‐min, and the CC current was set to ≈11 mA cm^−2^. Subsequent discharging was performed at 1 C (CC mode) for a complete single cycle. EIS study was conducted in Li||graphite half‐cell and graphite||NMC622 full‐cell formats using a multi‐purpose potentiostat (VMP‐300 and VSP‐300, Biologic) over a frequency range from 5 MHz to 50 mHz, with an AC amplitude of 10 mV. The DRT analysis was performed based on the EIS data. MATLAB (MathWorks, USA) was employed for the DRT calculations by exploiting both built‐in MATLAB add‐ons/functions and open‐source codes.^[^ ^68^ ^]^ The GITT experiments were conducted at 0.05 C, involving 15‐min galvanostatic pulses followed by 30‐min resting intervals using a battery tester.^[^ ^69^ ^]^

### Electrode Characterization

Field‐emission scanning electron microscopy (FE‐SEM; Su‐8020, Hitachi) was used to observe the morphology of the hybrid electrodes. An electrode resistance measurement system (RM2610, HIOKI) was used to measure the interfacial and bulk resistances of the electrode. To examine the spatial distribution of Li plating, the top surfaces of cycled anodes were analyzed by measuring C, F, and P Kα intensity using energy‐dispersive X‐ray (EDX) mapping. The XPS (ESCALAB 250Xi, Thermo Scientific) measurements were proposed to reveal the SEI component between the graphite||NMC622 full cell after precycling. A SAICAS was used to examine the electrode cohesion. The LIBS (J200, Applied Spectra) was used to analyze the SOC and binder distribution by extracting Li and Na.

### Computational Analysis

An electrochemical model was developed for the graphite||NMC622 full cell using COMSOL Multiphysics 6.1 (COMSOL Inc. USA). Based on Doyle and Newman's approach,^[^ ^68^ ^]^ the model was built in a P3D space with Cartesian dimensions x, y, and pseudo‐dimension *r* (see Figure S17a, Supporting Information). In this model, we incorporated the actual dimensions of the composite electrodes and the electrochemical properties of the SG and NG materials. The solid diffusion coefficient (*D_s_*) of each active material was determined from GITT measurements using the following Equation (1),^[^ ^70^, ^71^, ^72^, ^73^ ^]^

(1) $$D_{s}=\frac{4}{\pi \tau }\;{\left(\frac{r_{p}}{3}\right)}^{2}{\left(\frac{\Delta E_{s}}{\Delta E_{t}}\right)}^{2}$$

where Δ*E*_s_ and Δ*E*_t_ are the steady‐state and transient potential differences from the open circuit potential (OCP), respectively, *r*_p_ is the particle radius of the active materials determined through PSA analysis (Figures S2 and S8c,d, Supporting Information), and τ is the galvanostatic pulse duration time. Moreover, we calculated the exchange current density (*i*_0_) based on the EIS‐derived charge‐transfer resistance (*R*_ct_) using the following Equation (2) (Figure S21, Supporting Information),

(2) $$i_{0}=\frac{RT}{FSR_{\mathrm{ct}}}$$

where *R* is the gas constant (8.314 J mol^−1^ K^−1^), F is the Faraday constant (96485 C mol^−1^), and *S* is the active surface area. The *S* values of the active materials were obtained from the literature.^[^ ^74^, ^75^ ^]^ Additionally, the order of magnitude of the calculated *i*_0_ value was verified from the literature.^[^ ^76^ ^]^ In particular, the *D_s_* and *i*_0_ were defined as functions of the SOC to account for the temporal resistance variation of the SG and NG. The electrochemical properties of the electrolyte and cathode were obtained from the literature.^[^ ^77^, ^78^, ^79^, ^80^ ^]^ The solid and pore volume fractions of the cathode and anode were estimated based on the compositions and true densities of the electrode components. The effective parameters of Li^+^ diffusivity ($D_{l}^{\mathrm{eff}}$) and conductivity ($\sigma_{l}^{\mathrm{eff}}$) within the electrolyte were calculated by referring to the Bruggeman relation.^[^ ^81^, ^82^ ^]^ All governing equations of the model, including charge conservation, electroneutrality, mass conservation, Fick's law, Ohm's law, Butler–Volmer equation, and the respective boundary conditions, are listed in Table S2 (Supporting Information). All the parameters are summarized in Tables S3 and S4 (Supporting Information). For simulating the CC‐CV charging process at a 4 C‐rate, boundary conditions of 4.3 V and 0.4 C‐rate were additionally defined, with the final electrochemical model solved using the segregation step with the multifrontal massively parallel sparse direct solver (MUMPS).^[^ ^83^ ^]^ To visualize the temporal and spatial SOC distributions over a charging period, the SOC was derived by dividing the local *c*_s_ from the simulation results by the maximum Li concentration within the active material (*c*_s,max_) was theoretically calculated based on the specific capacity of NG and SG (360 and 340 mAh g^−1^ respectively) measured at the 0.1 C‐rate condition. For the time‐sequential overpotential (TSO) map, the local and temporal overpotentials were calculated as the difference between the potential and OCP.

## Conflict of Interest

The authors declare no conflict of interest.

## Supporting information

Supporting Information

## Data Availability

The data that support the findings of this study are available from the corresponding author upon reasonable request.

## References

[1] J. M. Tarascon , M. Armand , Nature 2001, 414, 359. 11713543 10.1038/35104644

[2] J. B. Goodenough , Y. Kim , Chem. Mater. 2010, 22, 587.

[3] Y. Liu , Y. Zhu , Y. Cui , Nat. Energy 2019, 4, 540.

[4] M. Li , J. Lu , Z. Chen , K. Amine , Adv. Mater. 2018, 30, 1800561. 10.1002/adma.201800561 29904941

[5] V. Etacheri , R. Marom , R. Elazari , G. Salitra , D. Aurbach , Energy Environ. Sci. 2011, 4, 3243.

[6] S.‐H. Park , P. J. King , R. Tian , C. S. Boland , J. Coelho , C. Zhang , P. McBean , N. McEvoy , M. P. Kremer , D. Daly , J. N. Coleman , V. Nicolosi , Nat. Energy 2019, 4, 560.

[7] H. Sun , L. Mei , J. Liang , Z. Zhao , C. Lee , H. Fei , M. Ding , J. Lau , M. Li , C. Wang , X. Xu , G. Hao , B. Papandrea , I. Shakir , B. Dunn , Y. Huang , X. Duan , Science 2017, 356, 599. 28495745 10.1126/science.aam5852

[8] M. Singh , J. Kaiser , H. Hahn , J. Electrochem. Soc. 2015, 162, A1196.

[9] K.‐Y. Park , J.‐W. Park , W. M. Seong , K. Yoon , T.‐H. Hwang , K.‐H. Ko , J.‐H. Han , Y. Jaedong , K. Kang , J. Power Sources 2020, 468, 228369.

[10] H.‐M. Cheng , F. Li , Science 2017, 356, 582. 28495714 10.1126/science.aan1472

[11] J. Asenbauer , T. Eisenmann , M. Kuenzel , A. Kazzazi , Z. Chen , D. Bresser , Sustain. Energy Fuels 2020, 4, 5387.

[12] M. Weiss , R. Ruess , J. Kasnatscheew , Y. Levartovsky , N. R. Levy , P. Minnmann , L. Stolz , T. Waldmann , M. Wohlfahrt‐Mehrens , D. Aurbach , M. Winter , Y. Ein‐Eli , J. Janek , Adv. Energy Mater. 2021, 11, 2101126.

[13] S. Li , K. Wang , G. Zhang , S. Li , Y. Xu , X. Zhang , X. Zhang , S. Zheng , X. Sun , Y. Ma , Adv. Funct. Mater. 2022, 32, 2200796.

[14] A. M. Colclasure , A. R. Dunlop , S. E. Trask , B. J. Polzin , A. N. Jansen , K. Smith , J. Electrochem. Soc. 2019, 166, A1412.

[15] K. G. Gallagher , S. E. Trask , C. Bauer , T. Woehrle , S. F. Lux , M. Tschech , P. Lamp , B. J. Polzin , S. Ha , B. Long , Q. Wu , W. Lu , D. W. Dees , A. N. Jansen , J. Electrochem. Soc. 2016, 163, A138.

[16] N. Ghanbari , T. Waldmann , M. Kasper , P. Axmann , M. Wohlfahrt‐Mehrens , J. Phys. Chem. C 2016, 120, 22225.

[17] T. Waldmann , M. Kasper , M. Wohlfahrt‐Mehrens , Electrochim. Acta 2015, 178, 525.

[18] T. Danner , M. Singh , S. Hein , J. Kaiser , H. Hahn , A. Latz , J. Power Sources 2016, 334, 191.

[19] Z. Du , D. L. Wood , C. Daniel , S. Kalnaus , J. Li , J. Appl. Electrochem. 2017, 47, 405.

[20] Z. Li , J. Huang , B. Yann Liaw , V. Metzler , J. Zhang , J. Power Sources 2014, 254, 168.

[21] T. Waldmann , B.‐I. Hogg , M. Wohlfahrt‐Mehrens , J. Power Sources 2018, 384, 107.

[22] K.‐H. Chen , M. J. Namkoong , V. Goel , C. Yang , S. Kazemiabnavi , S. M. Mortuza , E. Kazyak , J. Mazumder , K. Thornton , J. Sakamoto , N. P. Dasgupta , J. Power Sources 2020, 471, 228475.

[23] L. Kraft , J. B. Habedank , A. Frank , A. Rheinfeld , A. Jossen , J. Electrochem. Soc. 2020, 167, 013506.

[24] K.‐H. Chen , V. Goel , M. J. Namkoong , M. Wied , S. Müller , V. Wood , J. Sakamoto , K. Thornton , N. P. Dasgupta , Adv. Energy Mater. 2021, 11, 2003336.

[25] R. Diehm , J. Kumberg , C. Dörrer , M. Müller , W. Bauer , P. Scharfer , W. Schabel , Energy Technol. 2020, 8, 1901251.

[26] D. Liu , L.‐C. Chen , T.‐J. Liu , W.‐B. Chu , C. Tiu , Energy Technol. 2017, 5, 1235.

[27] M. A. Pimenta , G. Dresselhaus , M. S. Dresselhaus , L. G. Cançado , A. Jorio , R. Saito , Phys. Chem. Chem. Phys. 2007, 9, 1276. 17347700 10.1039/b613962k

[28] A. C. Ferrari , Solid State Commun. 2007, 143, 47.

[29] T. Placke , V. Siozios , R. Schmitz , S. F. Lux , P. Bieker , C. Colle , H. W. Meyer , S. Passerini , M. Winter , J. Power Sources 2012, 200, 83.

[30] J. Kim , A. J. Yun , K. Y. Sheem , B. Park , Nanomaterials 2021, 11, 1813. 34361199 10.3390/nano11071813 PMC8308424

[31] A. Guéguen , D. Streich , M. He , M. Mendez , F. F. Chesneau , P. Novák , E. J. Berg , J. Electrochem. Soc. 2016, 163, A1095.

[32] J. Henschel , C. Peschel , S. Klein , F. Horsthemke , M. Winter , S. Nowak , Angew. Chem., Int. Ed. 2020, 59, 6128. 10.1002/anie.202000727 PMC7187180 32012404

[33] Y. Yu , P. Karayaylali , Y. Katayama , L. Giordano , M. Gauthier , F. Maglia , R. Jung , I. Lund , Y. Shao‐Horn , J. Phys. Chem. C 2018, 122, 27368.

[34] T. Ishii , Y. Kaburagi , A. Yoshida , Y. Hishiyama , H. Oka , N. Setoyama , J. Ozaki , T. Kyotani , Carbon 2017, 125, 146.

[35] S. L. Glazier , J. Li , A. J. Louli , J. P. Allen , J. R. Dahn , J. Electrochem. Soc. 2017, 164, A3545.

[36] S. Niu , G. Zhu , K. Wu , H. Zheng , Chin. J. Chem. Eng. 2023, 56, 58.

[37] J. P. Schmidt , P. Berg , M. Schönleber , A. Weber , E. Ivers‐Tiffée , J. Power Sources 2013, 221, 70.

[38] B. Manikandan , V. Ramar , C. Yap , P. Balaya , J. Power Sources 2017, 361, 300.

[39] M. Yoshio , H. Wang , K. Fukuda , Angew. Chem., Int. Ed. 2003, 42, 4203. 10.1002/anie.200351203 14502736

[40] Y.‐S. Park , T.‐W. Lee , M.‐S. Shin , S.‐H. Lim , S.‐M. Lee , J. Electrochem. Soc. 2016, 163, A3078.

[41] J. Kumberg , M. Müller , R. Diehm , S. Spiegel , C. Wachsmann , W. Bauer , P. Scharfer , W. Schabel , Energy Technol. 2019, 7, 1900722.

[42] F. Font , B. Protas , G. Richardson , J. M. Foster , J. Power Sources 2018, 393, 177.

[43] H. Lee , C. Bak , M. Lim , H. An , S. Byun , Y. M. Lee , H. Lee , ACS Appl. Nano Mater. 2023, 6, 3128.

[44] Y. Jo , D. Jin , M. Lim , H. Lee , H. An , J. Seo , G. Kim , X. Ren , Y. M. Lee , H. Lee , Adv. Sci. 2023, 10, 2204812. 10.1002/advs.202204812 PMC9839847 36398609

[45] M. Baek , J. Kim , J. Jin , J. W. Choi , Nat. Commun. 2021, 12, 6807, 34815396 10.1038/s41467-021-27095-w PMC8611023

[46] S.‐M. Lee , J. Kim , J. Moon , K.‐N. Jung , J. H. Kim , G.‐J. Park , J.‐H. Choi , D. Y. Rhee , J.‐S. Kim , J.‐W. Lee , M.‐S. Park , Nat. Commun. 2021, 12, 39. 33397916 10.1038/s41467-020-20297-8 PMC7782533

[47] Z. Du , D. L. Wood , I. Belharouak , Electrochem. Commun. 2019, 103, 109.

[48] C. Sun , X. Ji , S. Weng , R. Li , X. Huang , C. Zhu , X. Xiao , T. Deng , L. Fan , L. Chen , X. Wang , C. Wang , X. Fan , Adv. Mater. 2022, 34, 2206020. 10.1002/adma.202206020 36067055

[49] T. Sudoh , K. Shigenobu , K. Dokko , M. Watanabe , K. Ueno , Phys. Chem. Chem. Phys. 2022, 24, 14269. 35667383 10.1039/d2cp01409b

[50] H. Gao , Q. Yan , J. Holoubek , Y. Yin , W. Bao , H. Liu , A. Baskin , M. Li , G. Cai , W. Li , D. Tran , P. Liu , J. Luo , Y. S. Meng , Z. Chen , Adv. Energy Mater. 2023, 13, 2202906.

[51] X. Xu , X. Yue , Y. Chen , Z. Liang , Angew. Chem., Int. Ed. 2023, 62, e202306963. 10.1002/anie.202306963 37384426

[52] J. Shi , N. Ehteshami , J. Ma , H. Zhang , H. Liu , X. Zhang , J. Li , E. Paillard , J. Power Sources 2019, 429, 67.

[53] H. Lee , H. An , H. Chang , M. Lee , S. Park , S. Lee , J. Kang , S. Byon , B. Koo , H. Lee , Y. M. Lee , J. Moon , S. Chae , H. Lee , Energy Storage Mater. 2023, 63, 102995.

[54] F. Lin , K. Zhao , Y. Liu , ACS Energy Lett. 2021, 6, 4065. 10.1021/acsenergylett.1c01868 PMC8593912 34805527

[55] C. Wei , S. Xia , H. Huang , Y. Mao , P. Pianetta , Y. Liu , Acc. Chem. Res. 2018, 51, 2484. 29889493 10.1021/acs.accounts.8b00123

[56] M.‐T. F. Rodrigues , K. Kalaga , S. E. Trask , D. W. Dees , I. A. Shkrob , D. P. Abraham , J. Electrochem. Soc. 2019, 166, A996.

[57] Q. Liu , C. Du , B. Shen , P. Zuo , X. Cheng , Y. Ma , G. Yin , Y. Gao , RSC Adv. 2016, 6, 88683.

[58] D. Tewari , Z. Liu , P. B. Balbuena , P. P. Mukherjee , J. Phys. Chem. C 2018, 122, 21097.

[59] X. Ma , X. Song , Y. Tang , E. Liu , C. Xu , C. Qi , Y. Li , J. Gao , Y. Li , J. Energy Chem. 2020, 49, 233.

[60] H. M. Hwang , D. Kim , K. Lee , H. Lee , Y. Luo , S. Oh , A. P. Tiwari , MRS Commun. 2020, 10, 25.

[61] Y. Wen , K. He , Y. Zhu , F. Han , Y. Xu , I. Matsuda , Y. Ishii , J. Cumings , C. Wang , Nat. Commun. 2014, 5, 4033. 24893716 10.1038/ncomms5033

[62] J. Huang , Q. Tang , W. Liao , G. Wang , W. Wei , C. Li , Ind. Eng. Chem. Res. 2017, 56, 5253.

[63] Q. Cheng , Y. Okamoto , N. Tamura , M. Tsuji , S. Maruyama , Y. Matsuo , Sci. Rep. 2017, 7, 14782. 29093496 10.1038/s41598-017-14504-8 PMC5665891

[64] J. Inamoto , S. Komiyama , S. Uchida , A. Inoo , Y. Matsuo , J. Phys. Chem. C 2022, 126, 16100.

[65] Z. Gao , N. Song , Y. Zhang , X. Li , Nano Lett. 2015, 15, 8194. 26588035 10.1021/acs.nanolett.5b03698

[66] N. Song , Z. Gao , Y. Zhang , X. Li , Nano Energy 2019, 58, 30.

[67] Y. Zhang , Z. Gao , X. Li , Small 2017, 13, 1701927. 10.1002/smll.201701927 28941060

[68] M. Doyle , J. Newman , A. S. Gozdz , C. N. Schmutz , J. M. Tarascon , J. Electrochem. Soc. 1996, 143, 1890.

[69] J. Kim , S. Park , S. Hwang , W.‐S. Yoon , J. Electrochem. Sci. Technol. 2022, 13, 19.

[70] W. Weppner , R. A. Huggins , J. Electrochem. Soc. 1977, 124, 1569.

[71] Z. Shen , L. Cao , C. D. Rahn , C.‐Y. Wang , J. Electrochem. Soc. 2013, 160, A1842.

[72] Y. Wei , J. Zheng , S. Cui , X. Song , Y. Su , W. Deng , Z. Wu , X. Wang , W. Wang , M. Rao , Y. Lin , C. Wang , K. Amine , F. Pan , J. Am. Chem. Soc. 2015, 137, 8364. 26098282 10.1021/jacs.5b04040

[73] S. Cui , Y. Wei , T. Liu , W. Deng , Z. Hu , Y. Su , H. Li , M. Li , H. Guo , Y. Duan , W. Wang , M. Rao , J. Zheng , X. Wang , F. Pan , Adv. Energy Mater. 2016, 6, 1501309.

[74] Q. Zhou , B. Wen , J. Zhang , F. Liu , X. Ouyang , Y. Liang , Z. Wu , Z. Xie , J. Cent. South Univ. 2023, 30, 665.

[75] C.‐L. Ma , Z.‐H. Hu , N.‐J. Song , Y. Zhao , Y.‐Z. Liu , H.‐Q. Wang , Rare Met. 2021, 40, 837.

[76] A. Swiderska‐Mocek , A. Lewandowski , J. Solid State Electrochem. 2017, 21, 1365.

[77] T. G. Zavalis , M. Behm , G. Lindbergh , J. Electrochem. Soc. 2012, 159, A848.

[78] A. Nyman , M. Behm , G. Lindbergh , Electrochim. Acta 2008, 53, 6356.

[79] O. Chaouachi , J.‐M. Réty , S. Génies , M. Chandesris , Y. Bultel , Electrochim. Acta 2021, 366, 137428.

[80] H. Lee , S. Yang , S. Kim , J. Song , J. Park , C.‐H. Doh , Y.‐C. Ha , T.‐S. Kwon , Y. M. Lee , Curr. Opin. Electrochem. 2022, 34, 100986.

[81] D. A. G. Bruggeman , Ann. Phys. 1935, 416, 636.

[82] B. Tjaden , S. J. Cooper , D. J. L. Brett , D. Kramer , P. R. Shearing , Curr. Opin. Chem. Eng. 2016, 12, 44.

[83] P. R. Amestoy , I. S. Duff , J. Y. L'Excellent , Comput. Methods Appl. Mech. Eng. 2000, 184, 501.

