# Supplementary material for: Sequential Effect of Dual‐Layered Hybrid Graphite Anodes on Electrode Utilization During Fast‐Charging Li‐Ion Batteries
Source: Adv Sci (Weinh). 2024 Jun 13;11(31):2403071. doi: 10.1002/advs.202403071 (PMC11336972; doi:10.1002/advs.202403071)
Supplement: Supplementary file 1 — Supporting Information [file ADVS-11-2403071-s001.docx]

Supporting Information

Sequential Effect of Dual-layered Hybrid Graphite Anodes on Electrode Utilization during Fast-charging Li-ion Batteries

*Jiwoong Kang^†^, Jaejin Lim^†^, Hyuntae Lee^†^, Seongsu Park, Cheol Bak, Yewon Shin, Hyeongguk An, Mingyu Lee, Minju Lee, Soyeon Lee, Byungjun Choi, Dongyoon Kang, Sujong Chae*, Yong Min Lee* and Hongkyung Lee**

J. Kang, J. Lim, H. Lee, C. Bak, Y. Shin, H. An, M. Lee, S. Lee, B. Choi, D. Kang, Prof. Y. M. Lee, Prof. H. Lee

Department of Energy Science and Engineering, Daegu Gyeongbuk Institute of Science and Technology (DGIST), 333 Techno Jungang-daero, Hyeonpung-eup, Dalseong-gun, Daegu 42988, Republic of Korea

*Email: hongkyung.lee@dgist.ac.kr (H. L.)

S. Park, Prof. S. Chae

Department of Industrial Chemistry, Pukyong National University, 45 Yongsoro, Busan 48513, Republic of Korea

*Email: schae@pknu.ac.kr (S. C.)

Prof. H. Lee

Energy Science and Engineering Center, DGIST, 333 Technojungang-daero, Daegu 42988, Republic of Korea

Prof. Y. M. Lee

Department of Chemical and Biomolecular Engineering, Yonsei University, 50 Yonsei-ro, Seodaemun-gu, Seoul 03722, Republic of Korea

*Email: yongmin@yonsei.ac.kr (Y. M. L.)

^†^These authors equally contributed to this work.

**
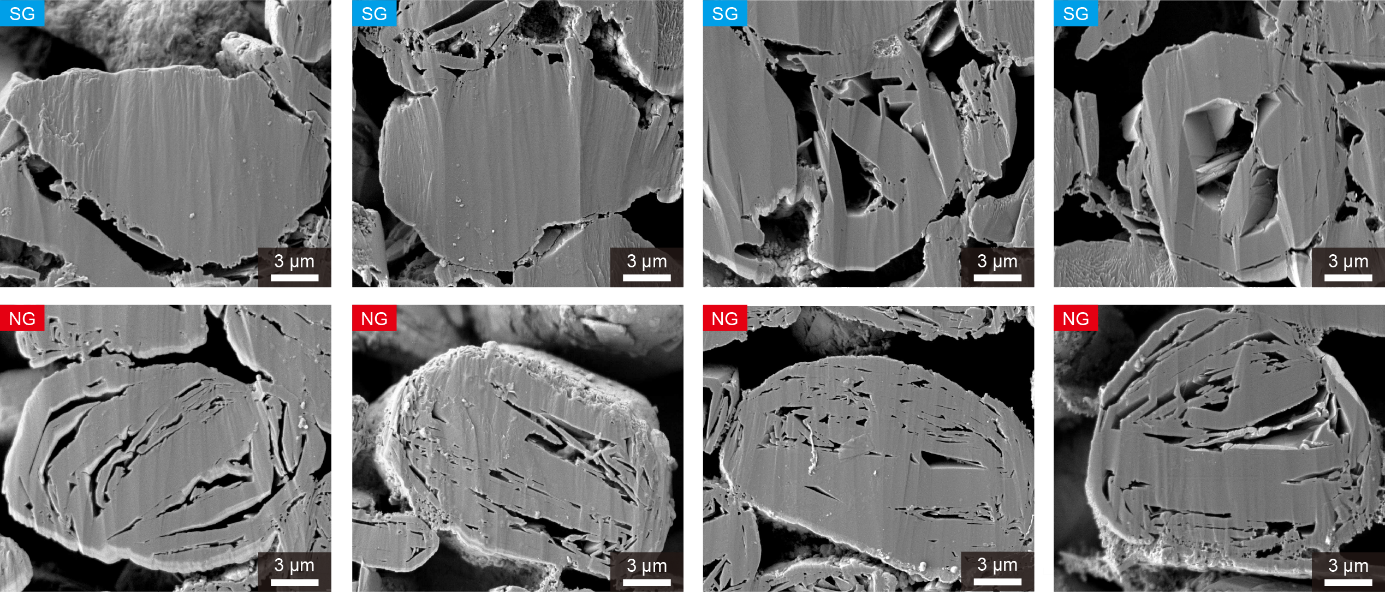
**

**Figure S1.** Cross-sectional SEM images of SG and NG particles.

**
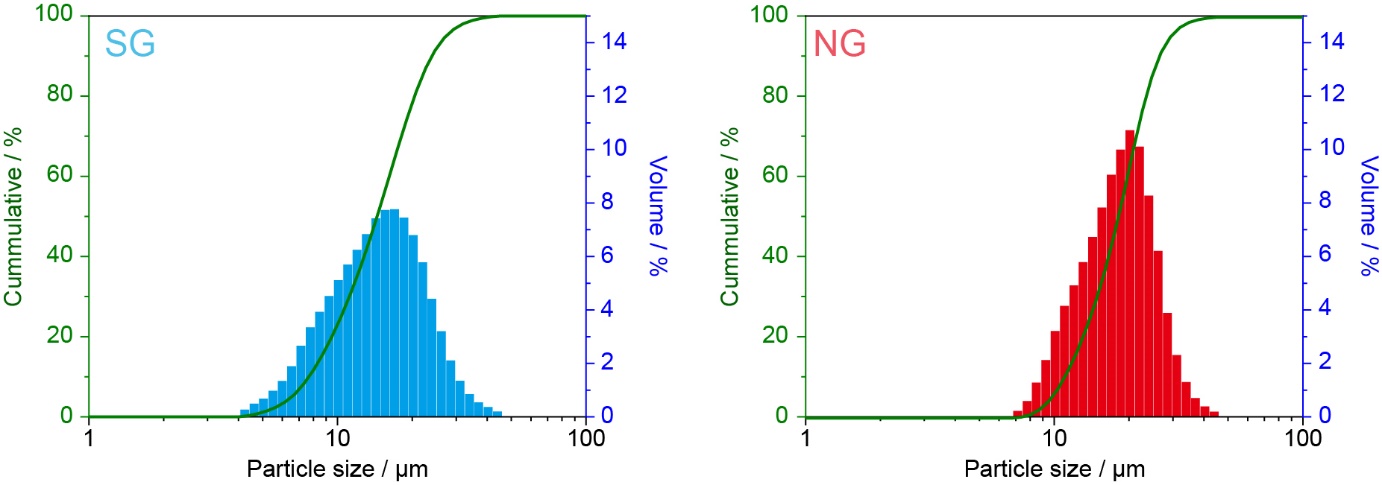
**

**Figure S2.** PSA histogram of SG and NG.

**
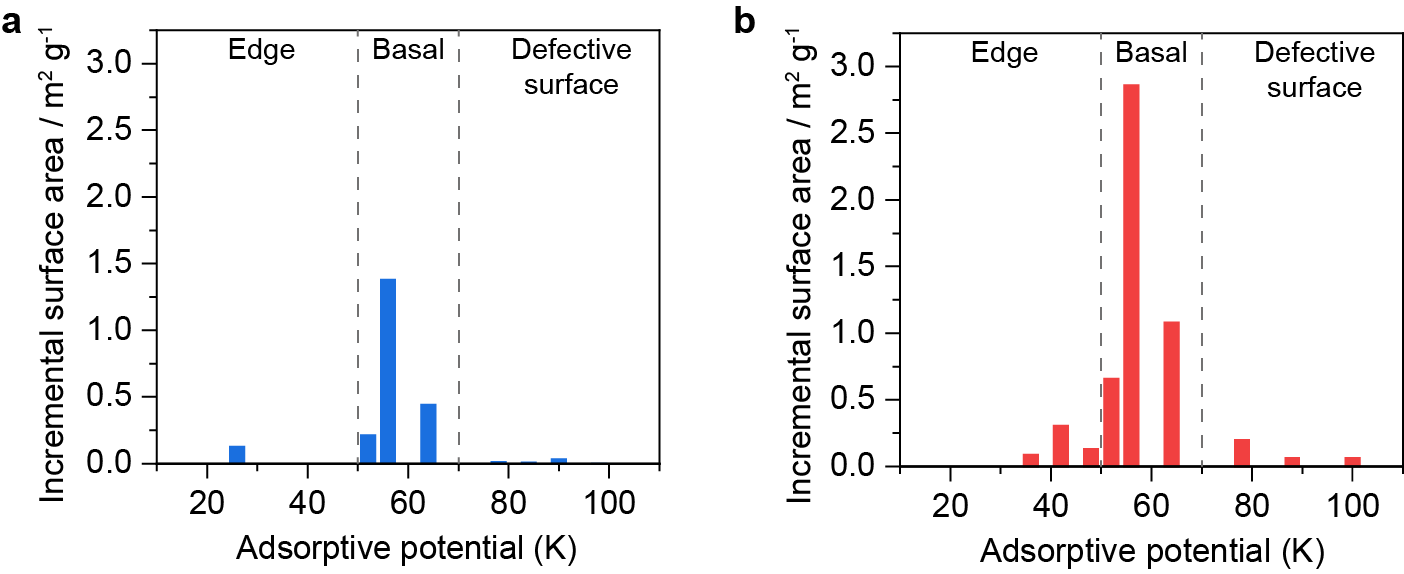
**

**Figure S3.** Incremental surface area vs. adsorptive potential plots of NG (a) and SG (b).

**
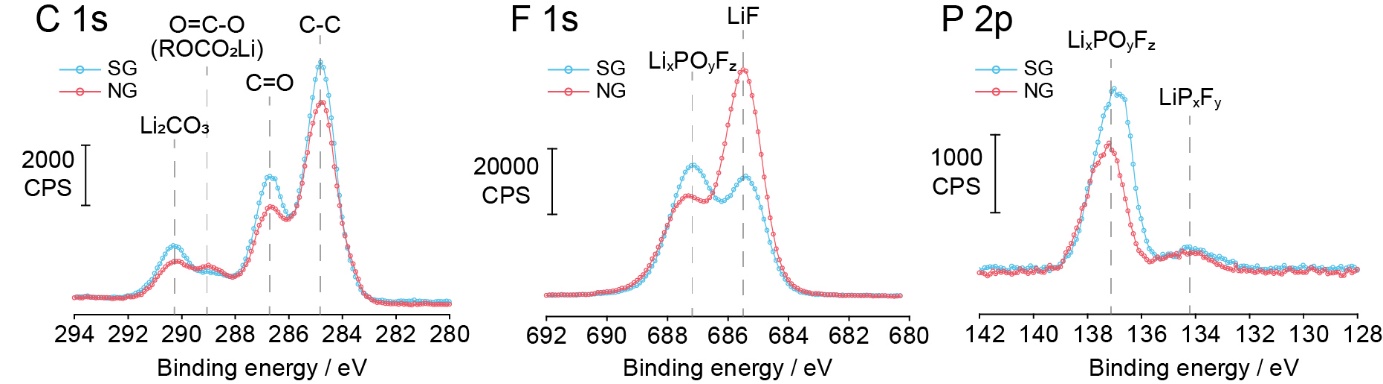
**

**Figure S4.** XPS C 1s, F 1s, and P 2p spectra for SEI formed at SG and NG after pre-cycle.


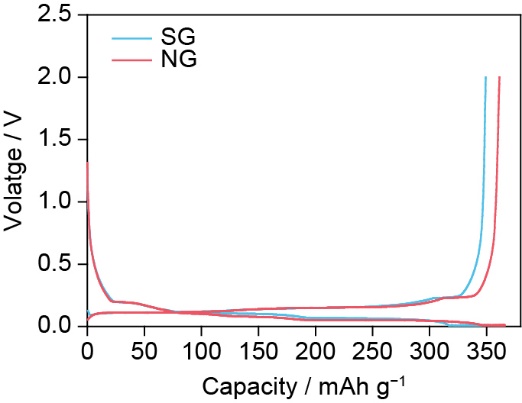


**Figure S5.** The voltage profile of Li||graphite half-cell after the formation cycle of SG and NG.


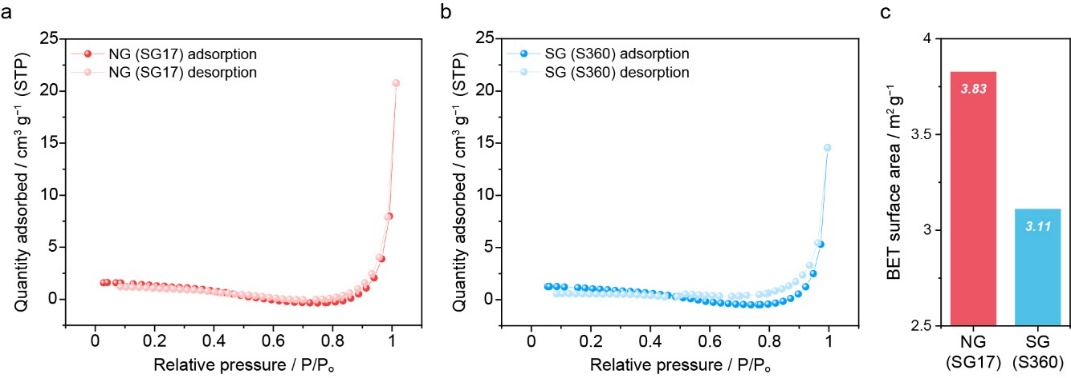


**Figure S6.** Nitrogen gas (N_2_) adsorption–desorption isotherm results: (a) NG and (b) SG. (c) Comparison of BET (Brunauer-Emmett-Teller) surface area of NG and SG.


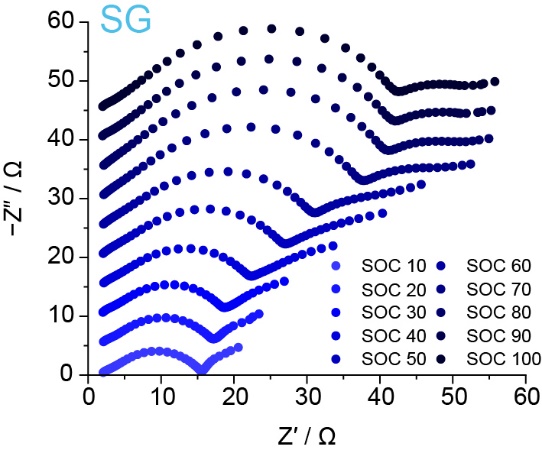

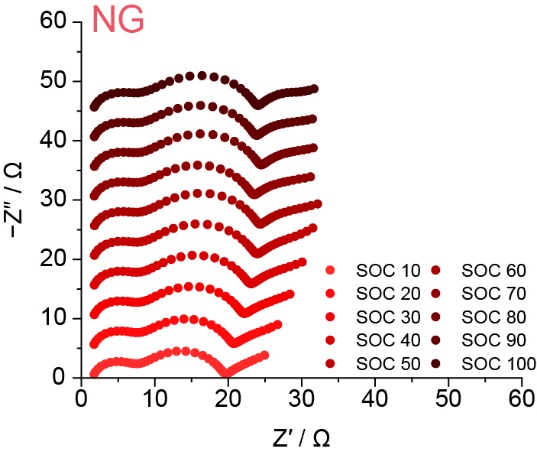


**Figure S7.** EIS spectra of SG and NG at varying the SOC.


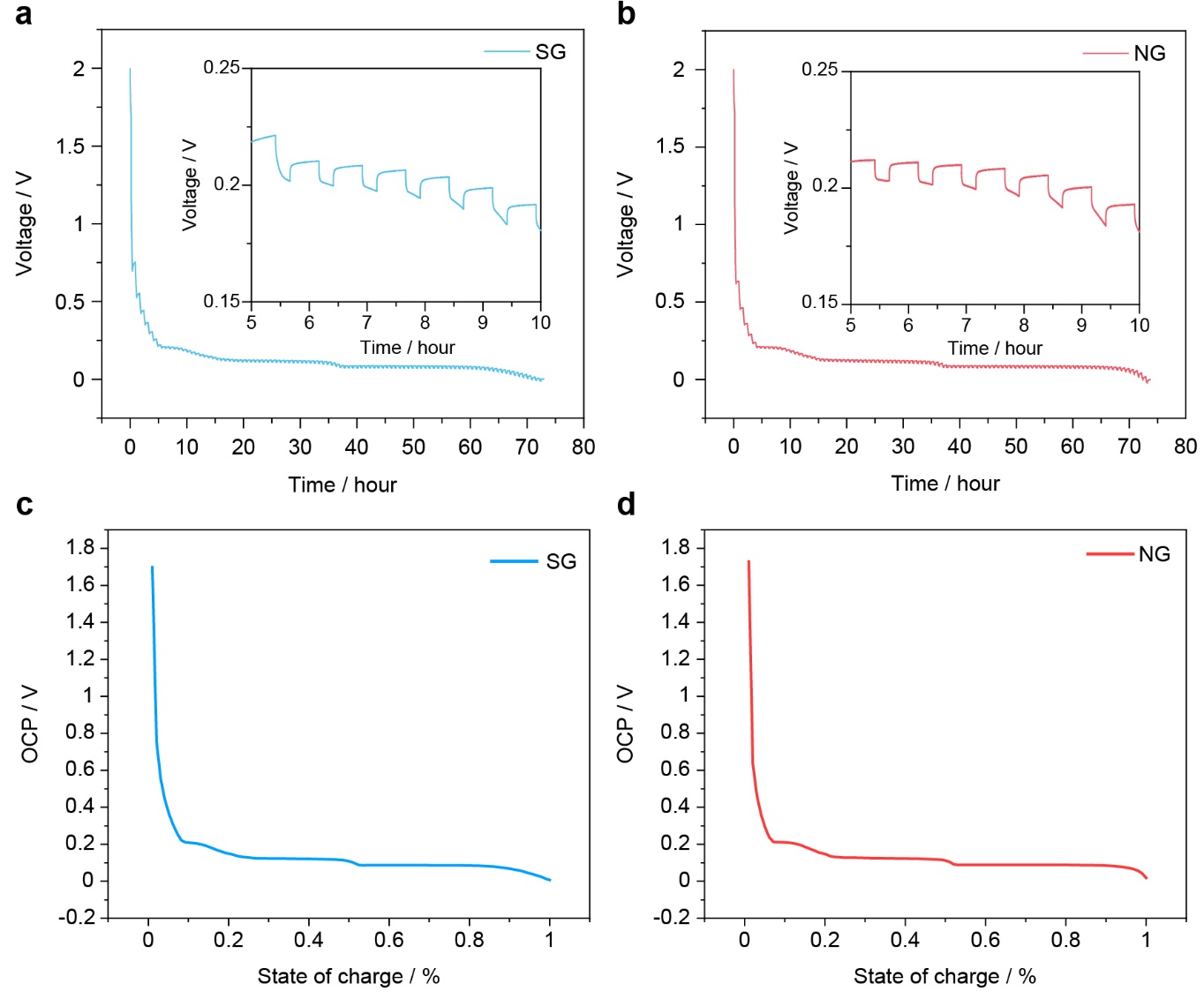
**Figure S8.** Time-voltage profile of GITT measurement (a) SG and (b) NG. The OCP curve from GITT measurement of (c) SG and (d) NG.


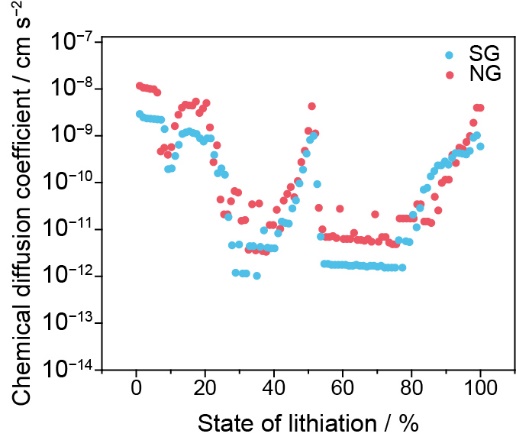


**Figure S9.** Comparison Li^+^ diffusion coefficient (*D*_Li+_) from GITT measurement of SG and NG


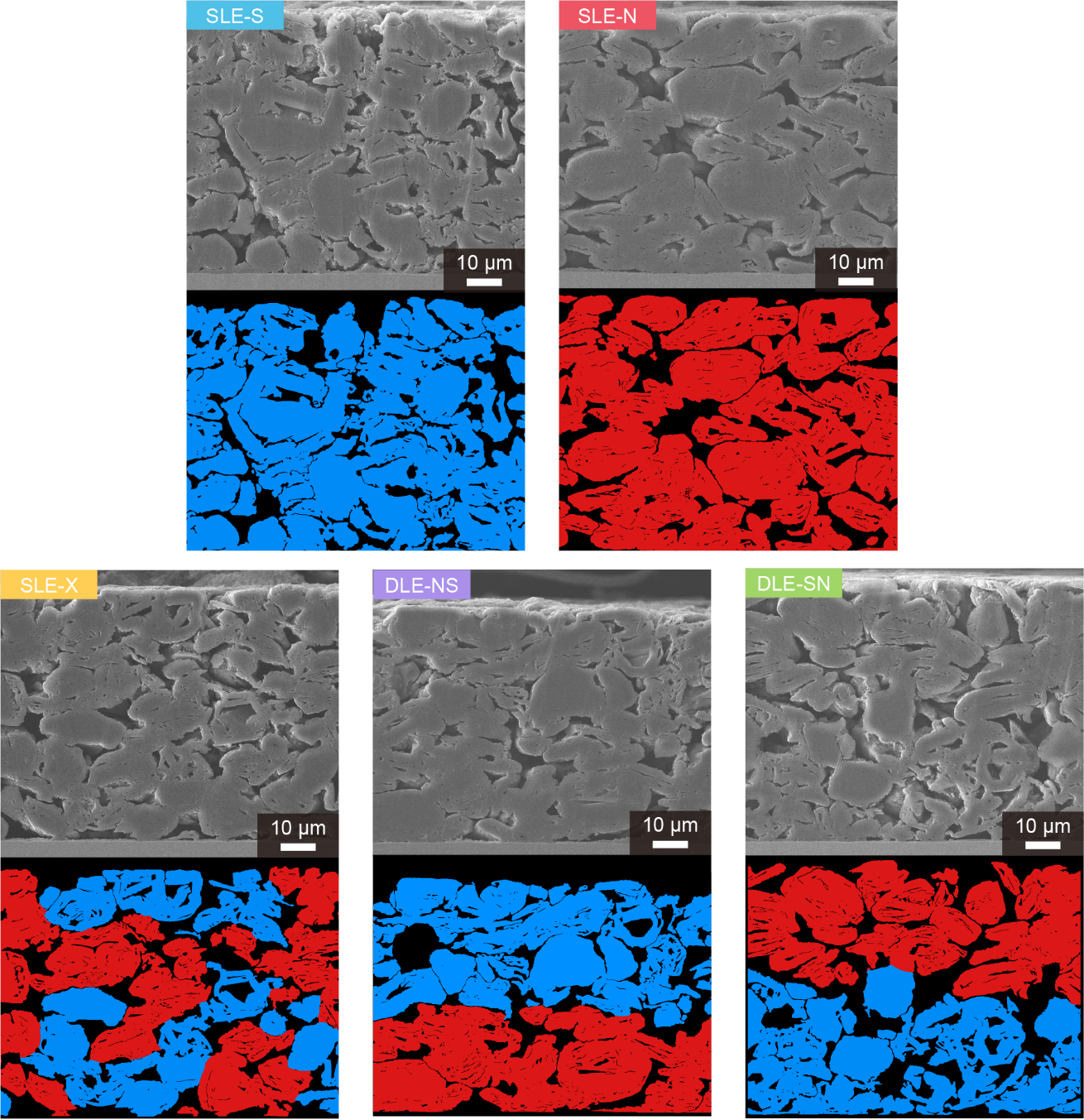


**Figure S10.** Cross-sectional SEM images of SLE-S, SLE-N, SLE-X, DLE-NS, and DLE-SN electrodes and their image segmentations.


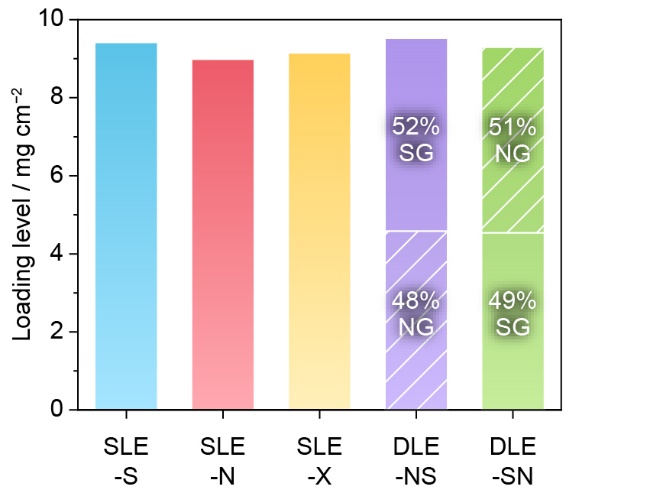


**Figure S11.** Comparison of the loading level of each electrode and that of each coating layer of DLEs


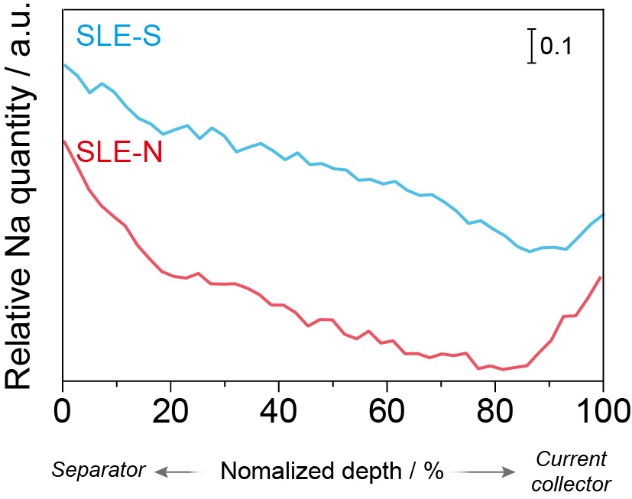


**Figure S12.** Depth profiling of relative Na^+^ counts measured by Na^+^-LIBS of SLE-S and SLE-N.


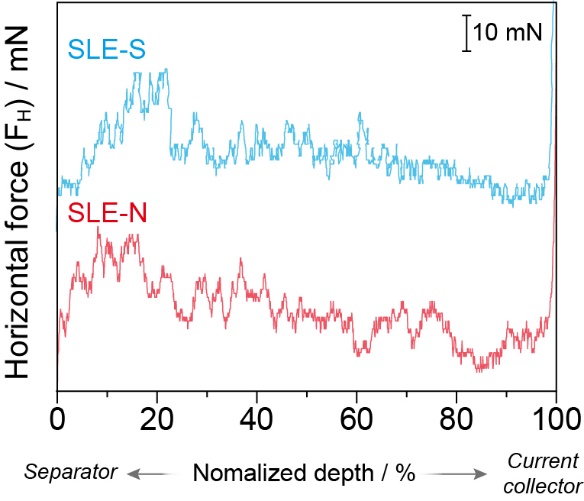


**Figure S13.** Depth profiling of horizontal forces (*F*_H_) measured by the SAICAS micro-blade of SLE-S and SLE-N

**
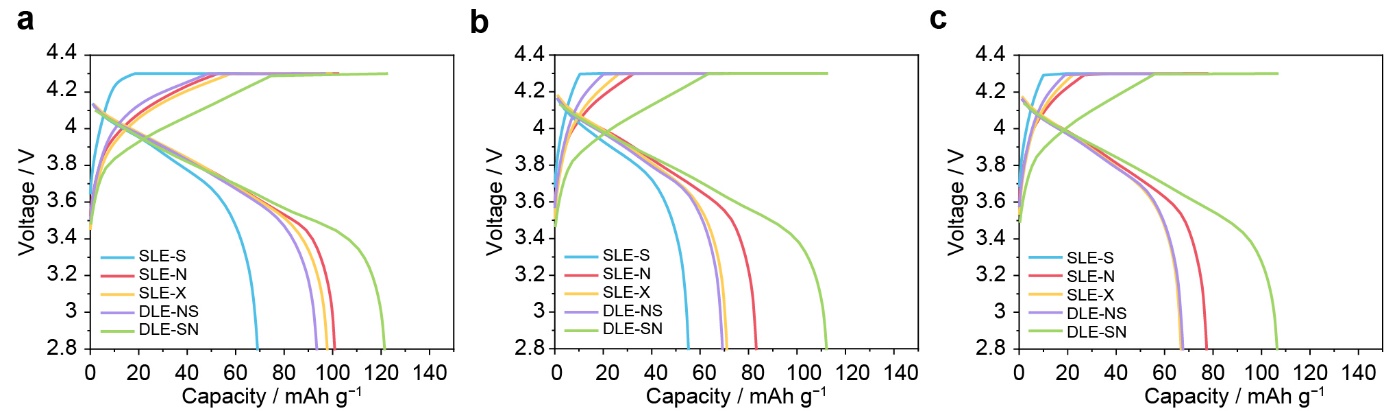
Figure S14.** Voltage profiles of graphite||NMC622 full cells (2.7 mAh cm^−2^) containing SLEs (SLE-S, SLE-N, and SLE-X) and DLEs (DLE-NS and DLE-SN) at (a) 25^th^ cycle (b) 100^th^ cycle (c) 200^th^ cycle under fast charging conditions (4 C, 15-min time cut-off).

**
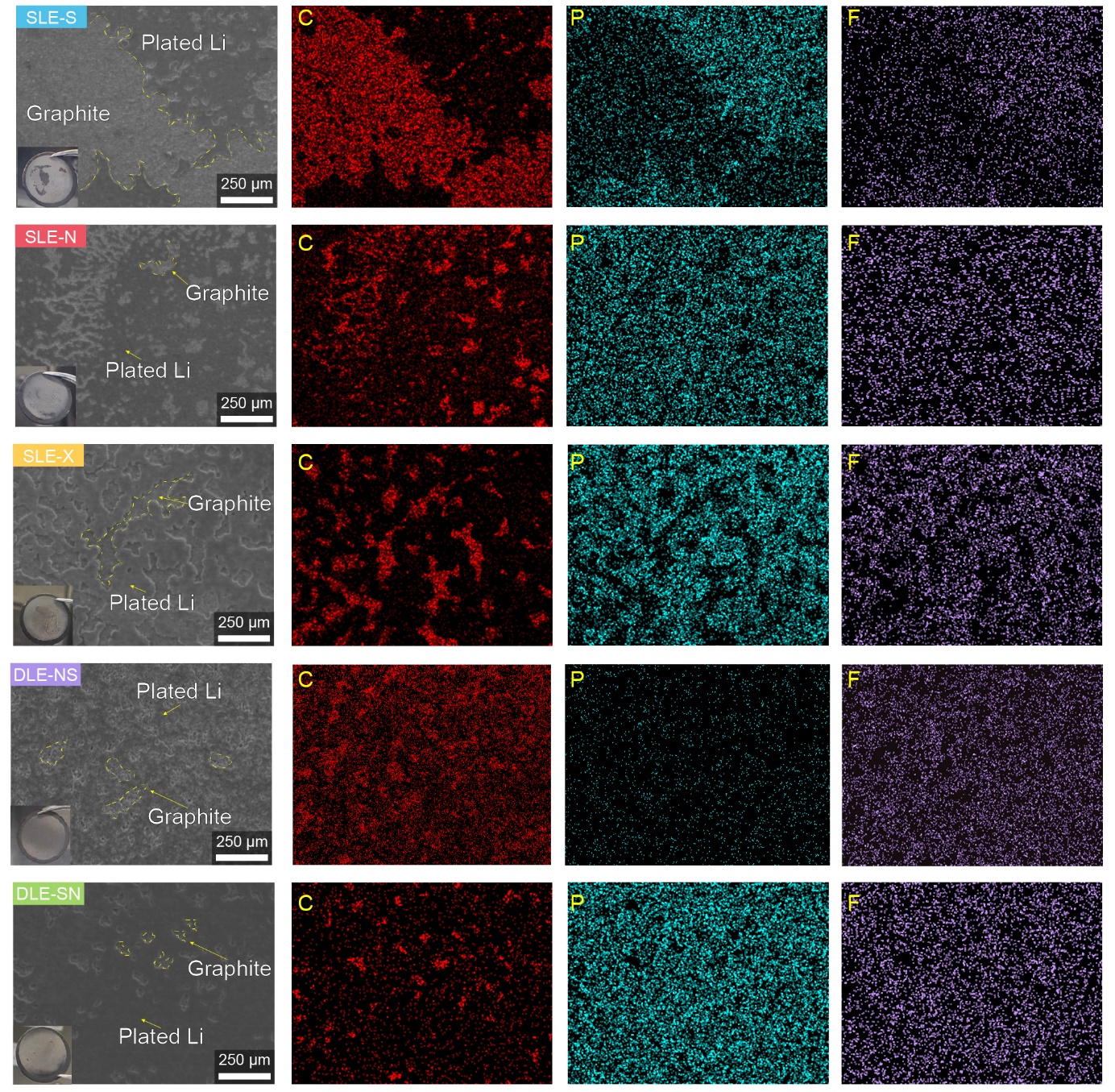
**

**Figure S15.** SEM/EDX mapping images and digital images of graphite anode harvested from graphite||NMC622 full cells after 10 cycles under fast-charging cycling (4 C): (a) SLE-S, (b) SLE-N, (c) SLE-X, (d) DLE-NS, and (e) DLE-SN.


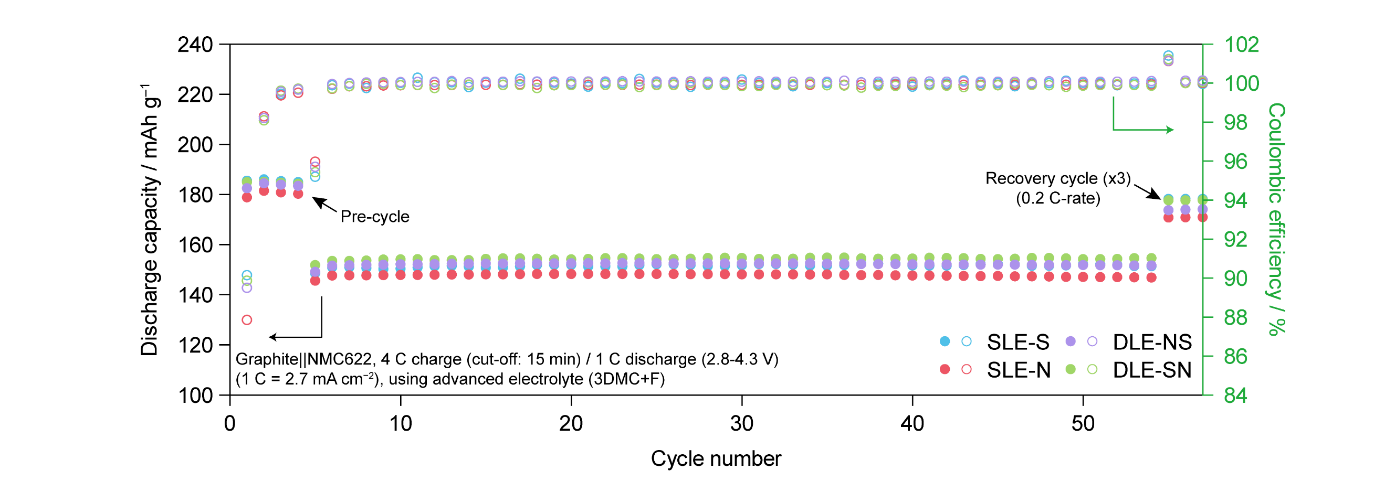


**Figure S16.** Cycle performances of graphite||NMC622 full cells (2.7 mAh cm^−2^, N/P = 1.1) at 4 C in the CC-CV charging mode (15-min time cut-off) with advanced electrolyte (3 M LiPF_6_ in DMC + 5 wt% FEC, which is denoted as 3DMC+F).


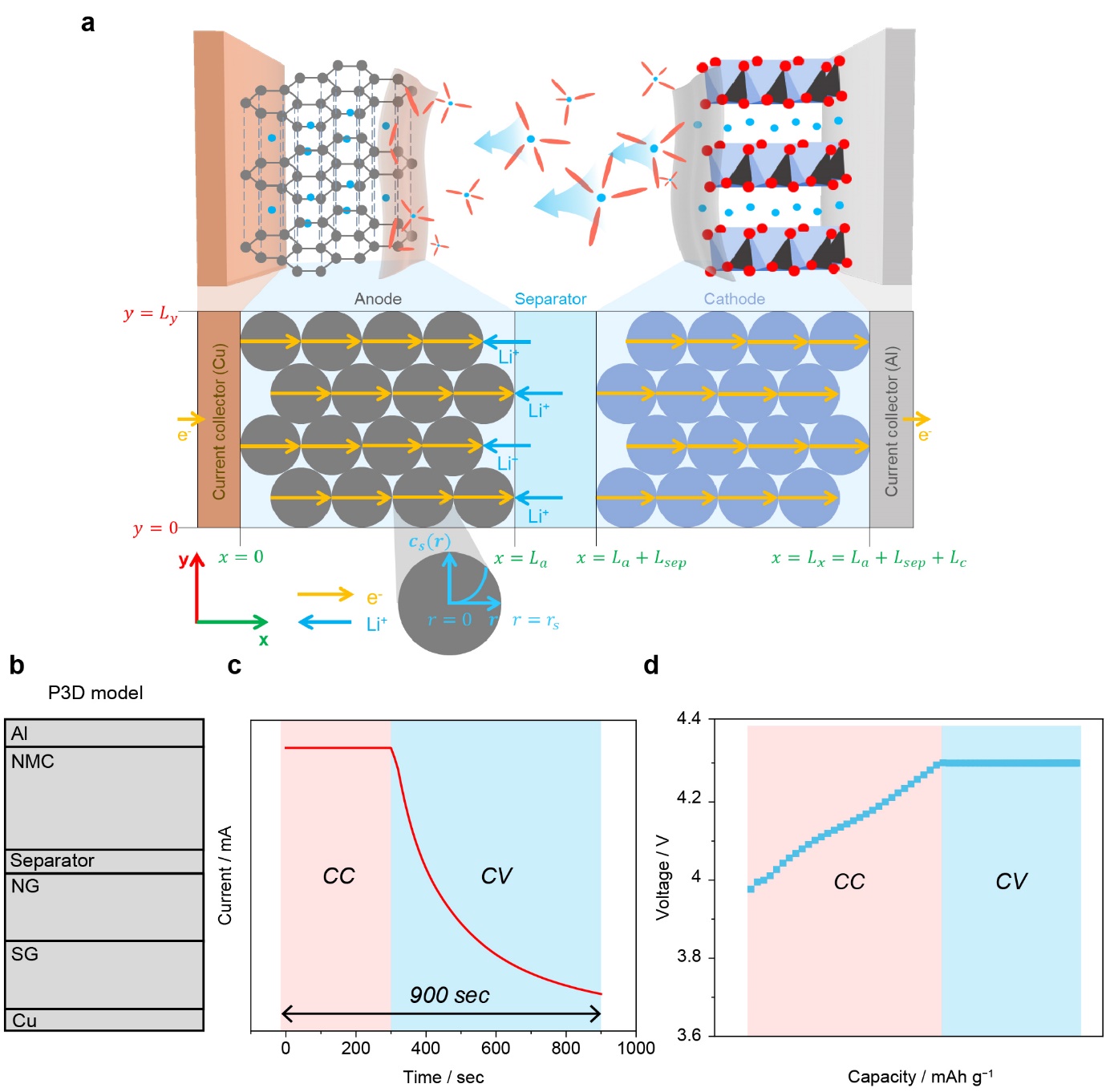


**Figure S17.** (a) Schematic of the P3D domain in the Newman’s model and the (b) full cell modeling geometry. The CC/CV model for the 4 C-rate fast charging simulation (c) current-time, (d) voltage-capacity curve.


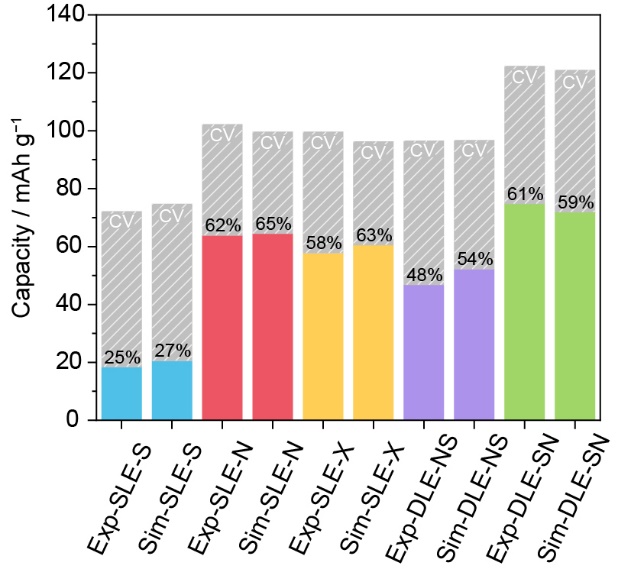


**Figure S18.** Comparative analysis of the CC and CV charging capacity after 25 cycles for each graphite anode at 4 C between experiment and simulation for the P3D model validation.


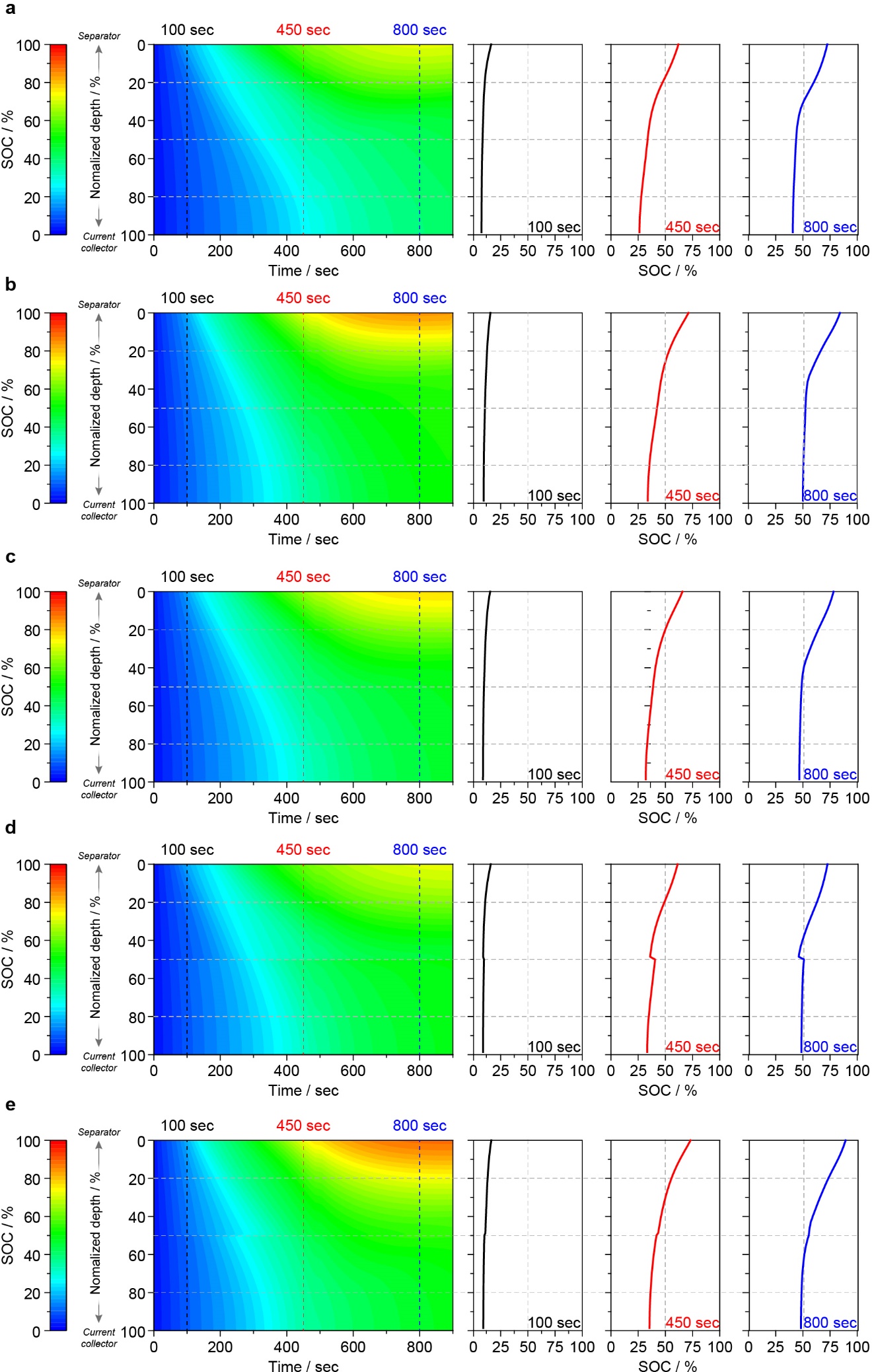


**Figure S19.** Time-resolved SOC distribution map of (a) SLE-S, (b) SLE-N, (c) SLE-X, (d) DLE-NS, and (e) DLE-SN


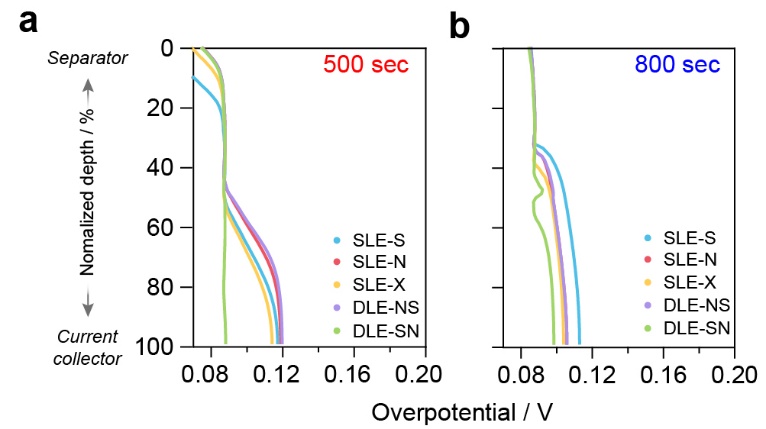


**Figure S20.** Line plots of time-sequential overpotential at (a) 500 sec, (b) 800 sec


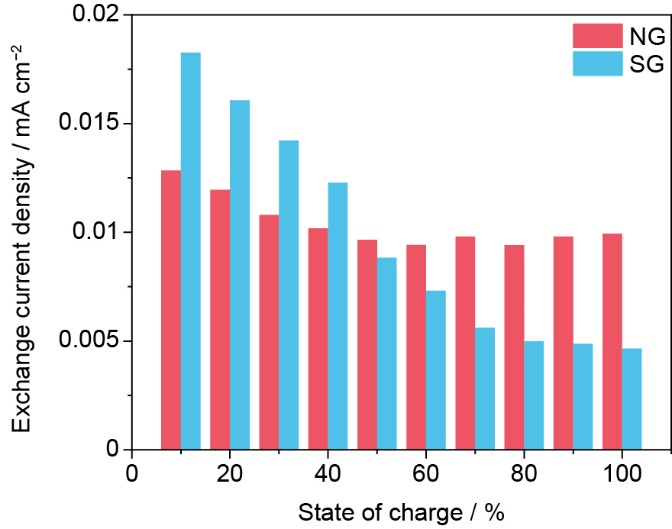


**Figure S21.** SOC-dependency of exchange current density estimated from EIS analysis.

**Table S1.** Total surface area, edge plane area, basal plane area, defective surface area, and the ratio of each surface area.

|  | **Total surface area (m^2^ g^-1^)** | **Edge plane (m^2^ g^-1^)** | **Basal plane (m^2^ g^-1^)** | **Defective surface (m^2^ g^-1^)** | **Edge plane (%)** | **Basal plane (%)** | **Defective surface (%)** |
| --- | --- | --- | --- | --- | --- | --- | --- |
| **SG** | 2.220 | 0.129 | 2.033 | 0.057 | 6 | 92 | 3 |
| **NG** | 5.456 | 0.531 | 4.599 | 0.326 | 10 | 84 | 6 |

**Table S2.** Governing equations and initial/boundary condition of P3D model

| **Electrochemical reaction** | **Governing equation** | **Domain** |
| --- | --- | --- |
| Diffusion and migration | $\frac{\partial c_{e}}{\partial t}=\nabla\cdot\left( D_{e}^{eff}\nabla c_{e} \right)-\nabla\cdot\frac{t_{+}\vec{j}_{e}}{F}$ | Electrolyte |
| Charge conservation  and electroneutrality | $\nabla\cdot\vec{j}_{e}=\nabla\cdot\left[ \sigma_{e}^{eff}(1-t_{+})\frac{2RT}{F}\left( 1+\frac{\partial lnf}{\partial lnc_{e}} \right)\nabla lnc_{e} \right]$ $-\nabla\cdot(\sigma_{e}^{eff}\nabla\phi_{e})$ | Electrolyte |
| Diffusion | $\frac{\partial c_{s}}{\partial t}=D_{s}^{eff}\left( \frac{\partial^{2}c_{s}}{\partial r^{2}}+\frac{2}{r}\frac{\partial c_{s}}{\partial r} \right)$ | Active material |
| Ohm’s law | $\nabla\cdot\vec{j}_{e}=-\nabla\cdot(\sigma_{s}\nabla\phi_{s})$ |  |
| Butler-Volmer equation | $i_{se}=2i_{0}\sinh\left[ (\phi_{s}-\phi_{e}-E_{eq})\frac{F}{2RT} \right]$  $\vec{j}_{e}\vec{n}={a_{s}i}_{se}$  $\vec{j}_{s}\vec{n}={a_{s}i}_{se}$ | Electrode/  Electrolyte  interface |

| **Initial and boundary conditions** | |
| --- | --- |
| Electrolyte mass transport | ${c_{e}\vert}_{t=0}=c_{e,ini}$  ${{\nabla c}_{e}\vert}_{x=0}={{\nabla c}_{e}\vert}_{x=L_{x}}=0$  ${{\nabla c}_{e}\vert}_{y=0}={{\nabla c}_{e}\vert}_{y=L_{y}}=0$  ${{{-D}_{e}^{eff}\nabla c}_{e}\vert}_{x=L_{a}}={-D}_{e}^{eff}{{\nabla c}_{e}\vert}_{x=L_{a}+L_{sep}}$ |
| Solid mass transport | ${c_{s}\vert}_{t=0}=c_{s,ini}$  $\left. \frac{\partial c_{s}}{\partial r} \right\vert_{r=0}=0$  $D_{s}^{eff}\left. \frac{\partial c_{s}}{\partial r} \right\vert_{r=r_{s}}=\frac{{a_{s}i}_{se}}{F}$ |
| Electrolyte electric filed | ${{\nabla\phi}_{e}\vert}_{x=0}={{\nabla\phi}_{e}\vert}_{x=L_{x}}=0$  ${{\nabla\phi}_{e}\vert}_{y=0}={{\nabla\phi}_{e}\vert}_{y=L_{y}}=0$  $\left( \sigma_{e}^{eff}\nabla^{2}\phi_{e}+FD_{e}^{eff}\nabla^{2}c_{e} \right)\vert_{x=L_{a}}=\left( \sigma_{e}^{eff}\nabla^{2}\phi_{e}+FD_{e}^{eff}\nabla^{2}c_{e} \right)\vert_{x=L_{a}+L_{sep}}$ |
| Solid electric field | ${{\nabla\phi}_{s}\vert}_{x=0}={{\nabla\phi}_{s}\vert}_{x=L_{x}}=0$  ${{\nabla\phi}_{s}\vert}_{y=0}={{\nabla\phi}_{s}\vert}_{y=L_{y}}=0$  ${{\nabla\phi}_{s}\vert}_{x=L_{a}}={{\nabla\phi}_{s}\vert}_{x=L_{a}+L_{sep}}$  $-\sigma_{s}^{eff}\nabla\phi_{s}\vert_{x=0}=\sigma_{s}^{eff}\nabla\phi_{s}\vert_{x=L_{x}}=\vec{j}_{s}$  $\eta=\phi_{s}-\phi_{e}-E_{eq}$ |

**Table S3.** P3D model parameters

| Symbol | Value | | Unit |
| --- | --- | --- | --- |
| $c_{e,ini}$ | 1.15 | | M, mol L^−1^ |
| $c_{max}$ | SG | 29,513^[a]^ | mol m^−3^ |
|  | NG | 30,525^[a]^ |  |
|  | NMC622 | 31,566^[a]^ |  |
| $E_{eq}$ | SG | Figure S13(a)^[b]^ | V |
|  | NG | Figure S13(b)^[b]^ |  |
|  | NMC622 | ^[1]^ |  |
| $\sigma_{e}$ | 0.93275 | | S m^−1^ |
| $\sigma_{s}$ | SG | 2390^[b]^ | S m^−1^ |
|  | NG | 2300^[b]^ |  |
|  | NMC622 | 1.76^[b]^ |  |
| $D_{e}$ | 3.8346×10^−10 [2]^ | | m^2^ sec^−1^ |
| $D_{s}$ | SG | Figure S5^[b]^ | m^2^ sec^−1^ |
|  | NG |  |  |
|  | NMC622 | 2ⅹ10^−13 [1]^ |  |
| $t_{+}$ | 0.250 ^[3]^ | | - |
| $i_{0}$ | SG | Figure S17^[b]^ | mA cm^−2^ |
|  | NG |  |  |
|  | NMC622 | 0.03 ^[4]^ |  |
| $L_{a}$ | 72.5^[b]^ | | μm |
| $L_{sep}$ | 13^[b]^ | | μm |
| $L_{c}$ | 51.2^[a]^ | | μm |
| $T$ | 298.15^[b]^ | | K |
| $R$ | 8.314 | | J mol^−1^ K^−1^ |
| $F$ | 96485 | | C mol^−1^ |

*[a] Theoretically calculated*

*[b] Experimentally measured*

**Table S4.** P3D full-cell design parameters

| Cell parameters |  | Unit | Values |
| --- | --- | --- | --- |
| Cathode  (NCM622) | Composition  (NCM622:PVDF: Super P) | - | 93:4:3 |
|  | Mass loading | mg cm^−2^ | 15.4 |
|  | Thickness | µm | 51.2 |
|  | Electrode density | g cm^−3^ | 3 |
|  | Al thickness | µm | 15 |
| Anode  (Gr) | Composition  (Graphite:CMC/SBR:Super P) | - | 94:1.5/1.5:3 |
|  | Mass loading | mg cm^−2^ | 9.4 |
|  | Thickness | µm | 72.5 |
|  | Electrode density | g cm^−3^ | 1.3 |
|  | Cu thickness | µm | 10 |
| N/P ratio |  | - | 1.2 |
| Separator |  | µm | 13 |
| Electrolyte |  | 1.15 M LiPF_6_ in EC/EMC (3:7, v/v) | |

**Table S5.** Glossary of symbols

| **Symbol** | **Description** | **Unit** |
| --- | --- | --- |
| $c_{e}$ | Lithium-ion concentration in electrolyte | mol m^−3^ |
| $c_{s}$ | Lithium-ion concentration in active material | mol m^−3^ |
| $c_{max}$ | Maximum lithium-ion concentration in active material | mol m^−3^ |
| $J_{e}$ | Current density in electrolyte | A cm^−2^ |
| $J_{s}$ | Current density in active material | A cm^−2^ |
| $i_{se}$ | Butler-Volmer interface current density | A cm^−2^ |
| $\phi_{e}$ | Electric potential of electrolyte | V |
| $\phi_{s}$ | Electric potential of active material | V |
| $E_{eq}$ | Equilibrium potential of active material (vs Li/Li^+^) | V |
| $\sigma_{e}$ | Ionic conductivity of electrolyte | S m^−1^ |
| $\sigma_{s}$ | Electronic conductivity of active material | S m^−1^ |
| $D_{e}$ | Lithium-ion diffusion coefficient of electrolyte | m^2^ s^−1^ |
| $D_{s}$ | Lithium-ion diffusion coefficient of active material | m^2^ s^−1^ |
| $t_{+}$ | Lithium-ion transference number of electrolyte | - |
| $i_{0}$ | Exchange current density | A cm^−2^ |
| $\eta$ | Overpotential | V |
| $L_{a}$ | Anode thickness | µm |
| $L_{sep}$ | Separator thickness | µm |
| $L_{c}$ | Cathode thickness | µm |
| $T$ | Temperature | K |
| $R$ | Universal gas constant (8.3143) | J mol^−1^K^−1^ |
| $F$ | Faraday constant (96,487) | C mol^−1^ |
| $ini$ | Initial state | - |
| $eff$ | Effective parameter | - |

References

[1] O. Chaouachi, J.-M. Réty, S. Génies, M. Chandesris, Y. Bultel, *Electrochim. Acta* **2021**, *366*, 137428.

[2] T. G. Zavalis, M. Behm, G. Lindbergh, *J. Electrochem. Soc.* **2012**, *159*, A848.

[3] A. Nyman, M. Behm, G. Lindbergh, *Electrochim. Acta* **2008**, *53*, 6356.

[4] H. Lee, S. Yang, S. Kim, J. Song, J. Park, C.-H. Doh, Y.-C. Ha, T.-S. Kwon, Y. M. Lee, *Curr. Opin. Electrochem.* **2022**, *34*, 100986.
